# Intrinsic auxetic piezoelectricity in bulk ferroelectrics

**DOI:** 10.1093/nsr/nwag295

**Published:** 2026-05-20

**Authors:** Zhi Tan, He-Meng Sun, Wei Shi, Shangyi Guan, Hui Zhang, Laiming Jiang, Qiang Chen, Jie Xing, Jianguo Zhu, Ming-Min Yang

**Affiliations:** College of Materials Science and Engineering, Sichuan University, Chengdu 610065, China; School of Emerging Technology, University of Science and Technology of China, Hefei 230026, China; Hefei National Laboratory, Hefei 230088, China; College of Materials Science and Engineering, Sichuan University, Chengdu 610065, China; College of Materials Science and Engineering, Sichuan University, Chengdu 610065, China; Shaanxi Key Laboratory of High-Orbits-Electron Materials and Protection Technology for Aerospace, School of Advanced Materials and Nanotechnology, Xidian University, Xi’an 710126, China; College of Materials Science and Engineering, Sichuan University, Chengdu 610065, China; College of Materials Science and Engineering, Sichuan University, Chengdu 610065, China; College of Materials Science and Engineering, Sichuan University, Chengdu 610065, China; College of Materials Science and Engineering, Sichuan University, Chengdu 610065, China; School of Emerging Technology, University of Science and Technology of China, Hefei 230026, China; Hefei National Laboratory, Hefei 230088, China

**Keywords:** Aurivillius ferroelectrics, piezoelectric ceramics, transverse piezoelectricity, first-principles calculations, electromechanical coupling

## Abstract

Piezoelectric materials deform under an electric field, typically exhibiting opposite signs in their longitudinal and transverse piezoelectric coefficients. Achieving the same sign for both coefficients enables the ‘auxetic piezoelectric effect’, where the material expands or contracts simultaneously in all directions. Although this unconventional effect has recently been observed in carefully engineered single-crystal heterostructures along specific crystallographic directions, a low-cost intrinsic bulk polycrystalline material that combines auxetic response with high-temperature stability has remained elusive. Here, we first predict and experimentally demonstrate a catalog of bulk materials exhibiting this counterintuitive intrinsic auxetic piezoelectric effect, namely the Aurivillius ferroelectrics. Theoretical analyses reveal that this effect originates from the relative sliding deformation between differently charged structural units within their layered architecture, and that it remains robust over a broad temperature range (5–1000 K). Furthermore, we show that the auxetic nature of bulk Aurivillius ceramics can be exploited to enhance the effective longitudinal piezoelectric response by an order of magnitude, achieving coexistence of strong piezoelectricity and high operating temperature. This work establishes Aurivillius ferroelectrics as a platform for auxetic piezoelectrics and opens avenues for advanced electromechanical devices.

## Teaser

A counter-intuitive auxetic piezoelectric effect is discovered in intrinsic bulk ferroelectrics, where the same-sign longitudinal and transverse piezoelectric coefficients give rise to simultaneous expansion or contraction along orthogonal directions under an external electric field.

## INTRODUCTION

The piezoelectric effect allows the inter-conversion between mechanical and electrical energy, leading to widespread applications, such as acceleration sensing [1,2], precision actuation [3,4], ultrasonic medical imaging [5,6] and emerging piezoelectric energy harvesting [7]. Deliberately controlling the value of piezoelectric coefficients *d_ij_* by methods such as cutting crystals along certain crystallographic orientations [8] or epitaxial growth [9], has constituted a crucial strategy to either enhance electromechanical coupling along desired directions or to diminish parasitic response that causes energy loss. Nevertheless, the sign of transverse piezoelectric coefficients (*d*_31_, *d*_32_) usually stays in the opposite sign with respect to that of the longitudinal one (i.e. *d*_33_). Recent demonstration of the auxetic piezoelectric effect represents a breakthrough in this regard, in which *d*_33_, *d*_31_ and *d*_32_ are of the same sign [10,11]. Notably, auxetic piezoelectricity is fundamentally different from conventional structural auxeticity, which refers to a purely mechanical effect associated with a negative Poisson’s ratio. In contrast, materials exhibiting auxetic piezoelectricity may still possess a positive Poisson’s ratio, while their piezoelectric response reflects an unusual electromechanical coupling behavior, in which an applied electric field can simultaneously induce same-signed strains in both the longitudinal and transverse directions. The auxetic piezoelectric effect offers further feasibility to engineer piezoelectric properties, for example, which can be employed to enhance the longitudinal piezoelectric sensitivity at the case of fixed transverse strain [12–14].

The auxetic piezoelectric effect was first predicted by J. Liu and S. Liu *et al.* in 2020. Their calculations showed the orthorhombic HfO_2_ and quasi-2D ternary compounds *A*Sn*X* (*A* = Na, K and *X* = N, P, space group *P*6_3_*mc*) exhibited the auxetic piezoelectric behavior [11], attributed to the negative *d*_33_ arose from the dominance of the negative internal-strain contribution over the positive clamped-ion contribution. This effect was later experimentally observed in (111)-oriented single-crystal heterostructures, including Schottky junctions (e.g. Au/Nb : SrTiO_3_) and tricolor superlattices, where piezoelectricity arises from interfacial asymmetry rather than the intrinsic crystalline structure [10,15]. In these heterostructures, the interface piezoelectric effect is determined by the synergy of chemical potential gradient and electrostriction effect. Both sign and magnitude of the electrostriction coefficients can be tuned by crystallographic orientations, leading to the auxetic piezoelectric effect. However, despite these advances, the effect has only been realized in single-crystal heterostructures with highly engineered interfaces and specific orientations, resulting in extremely high fabrication costs and limited practical applicability. Whether an intrinsic ferroelectric polycrystalline material can exhibit a robust auxetic piezoelectric effect remains an open question. Demonstrating such a material would not only deepen our understanding of electromechanical coupling in bulk ferroelectrics but also enable broader technological exploitation of this unconventional response. To this end, through a combination of DFT calculations and experimental characterization, we demonstrate that a first catalog of bulk materials termed bismuth layered ferroelectrics with Aurivillius structure exhibit the intrinsic auxetic piezoelectricity in both single crystal and ceramic forms, arising from crystal symmetry and collective atomic response rather than the engineered interfaces or defects.

**Figure 1.**
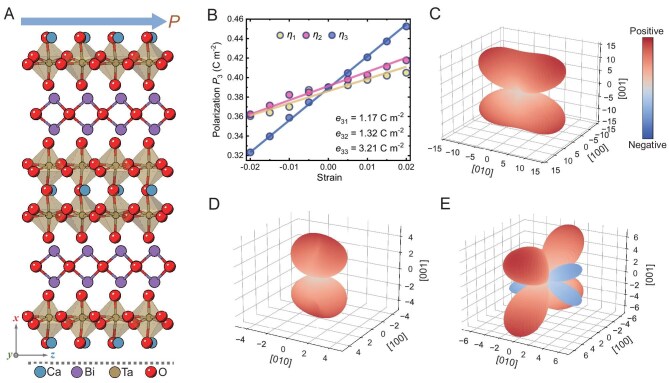
Crystal structure and calculated piezoelectric coefficients of CaBi_2_Ta_2_O_9_. (A) Crystal structure of ferroelectric *Cmc*2_1_ CaBi_2_Ta_2_O_9_ with Bi_2_O_2_ layer and perovskite block alternatively intergrown along the *x* direction. (B) Dependence of polarization *P*_3_ on uniaxial strain. (C–E) Three-dimensional spherical polar plots of the crystallographic-orientation-dependent (C) longitudinal piezoelectric coefficient *d*_33_, transverse piezoelectric coefficients (D) *d*_31_ and (E) *d*_32_ at *φ* = 0°.

Bismuth-layered structure oxides, such as Bi_4_Ti_3_O_12_, CaBi_2_Ta_2_O_9_, Bi_2_WO_6_ and related compounds, are a type of ferroelectric composed of perovskite-like blocks separated by (Bi_2_O_2_)^2+^ sheets (Fig. 1A and Table S1) [16,17]. Due to their unique structure, these materials exhibit novel properties including high Curie temperature, high resistivity and high polarization stability even at nanometer thicknesses [18–22]. Here, we take CaBi_2_Ta_2_O_9_ as an example to show the auxetic piezoelectric feature of these materials in both crystal and ceramic forms. To theoretically calculate its piezoelectric coefficients, we employ first-principles calculations based on density functional theory to evaluate the polarization evolution of ferroelectric CaBi_2_Ta_2_O_9_ under uniaxial strain. The results show that the polarization *P*_3_ increases linearly with the uniaxial strains *η*_1_, *η*_2_, and *η*_3_, respectively, giving positive piezoelectric stress constants *e*_31_ = 1.17 C m^−2^, *e*_32_ = 1.32 C m^−2^, and *e*_33_ = 3.21 C m^−2^ (see Fig. 1B, verified by Fig. S1). The piezoelectric strain constants are further calculated using the piezoelectric stress tensor and the elastic tensor (see Tables S2–S4) obtained by density functional perturbation theory (DFPT). The results show the *d*_31_, *d*_32_, and *d*_33_ of CaBi_2_Ta_2_O_9_ crystal are 5.11, 2.86, and 14.24 pC N^−1^, respectively. All transverse and longitudinal piezoelectric strain constants are positive, corresponding to the desired auxetic piezoelectricity.

To gain further insight into its piezoelectric behavior, we obtained the three-dimensional spherical polar plot of *d*_31_, *d*_32_, and *d*_33_ using the tensor transformation, which can be described by Euler angles *ψ,θ, φ* in the ZXZ convention (Detailed materials and methods and Fig. S2 in Supplementatry Materials). In the case of rotation angle *φ* = 0, the effective *d*_33_ and *d*_31_ remains positive at all directions in Fig. 1C and D, and the effective *d*_32_ is always positive in the *y*-*z* plane in Fig. 1E. Angle (*θ*)-dependent piezoelectric coefficients *d*_31_, *d*_32_ and *d*_33_ in CaBi_2_Ta_2_O_9_ crystal at different *ψ* and *φ* are plotted in Figs S3–S4, showing that the *d*_31_, *d*_32_ and *d*_33_ can be positive simultaneously in the low-*θ* region. It should be noted that the CaBi_2_Ta_2_O_9_ is a ferroelectric material and its polarization can be aligned to the external electric field. In our case, the longitudinal direction, i.e. the out-of-plane direction of the CaBi_2_Ta_2_O_9_ ceramic disk, corresponds to the direction of the applied electric field during the poling process. During poling, the external electric field drives the ferroelectric domains to align preferentially along the field direction. As a result, the polarization vectors of these domains become as closely aligned as possible with the applied electric field (i.e. domains with smaller *θ* are energetically favored). After removal of the electric field, these domains largely retain their reoriented states, allowing the ceramic to maintain a macroscopic remanent polarization. This implies that, in poled ferroelectric ceramics, most domains remain within the low-*θ* region (see Fig. S5). Under these conditions, the macroscopic piezoelectric coefficients of the ceramics can be reasonably approximated as the average over all domains. Thus, it is likely that CaBi_2_Ta_2_O_9_ is also auxetic piezoelectricity in its ceramic form. The piezoelectric constants of CaBi_2_Ta_2_O_9_ ceramics can be obtained from that of single crystal using the developed orientational average method [23], and it is found that the *d*_31_ and *d*_33_ of CaBi_2_Ta_2_O_9_ ceramic are both positive with magnitude of 3.44 and 8.82 pC N^−1^, respectively, which are of auxetic nature (Table S5). Further molecular dynamics simulations show that the auxetic piezoelectric effect in CaBi_2_Ta_2_O_9_ ceramic is robust and can be preserved up to the Curie temperature, which even can be enhanced with increasing temperature to 800 K (Figs S6 and S7, Tables S6 and S7).

**Figure 2.**
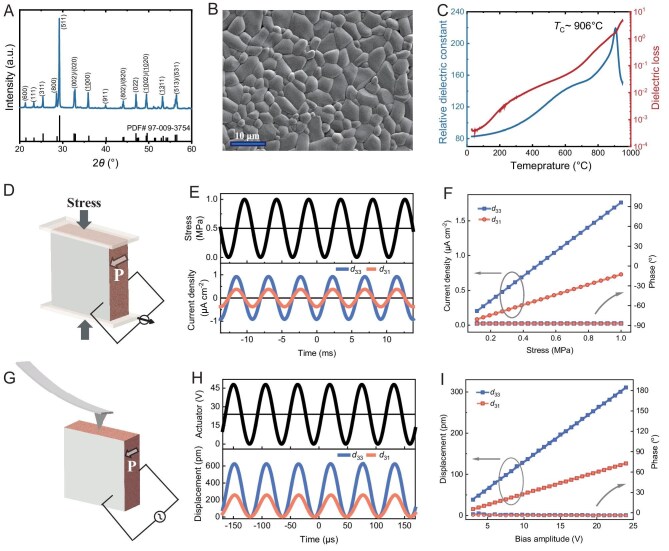
Experimental demonstration of the auxetic piezoelectric effect in CaBi_2_Ta_2_O_9_ ceramics. (A) X-ray diffraction (XRD) pattern of CaBi_2_Ta_2_O_9_ ceramics. (B) Surface scanning electron microscopy (SEM) photograph of CaBi_2_Ta_2_O_9_ ceramics. (C) Dielectric constant vs. temperature of CaBi_2_Ta_2_O_9_ ceramics, measured at 1 MHz. (D) Schematic of the measurement geometry to characterize the direct piezoelectric coefficients *d*_31_. (E) Waveform of applied sinusoidal compressive stress (top panel), and the induced current density (bottom panel) for Ag/CaBi_2_Ta_2_O_9_/Ag capacitors, with stress applied parallel (*d*_33_) and perpendicular (*d*_31_) to the direction of polarization. (F) Stress-dependent short-circuit current density induced by piezoelectric *d*_33_ and *d*_31_ with the measurement frequency of 217.7 Hz. (G) Schematic of the measurement geometry to characterize the converse piezoelectric coefficients *d*_31_. (H) Waveform of the applied sinusoidal bias voltage (top panel), and the induced displacement waveform (bottom panel) for Ag/CaBi_2_Ta_2_O_9_/Ag capacitors, with the induced displacement parallel (*d*_33_) and perpendicular (*d*_31_) to the direction of polarization. (I) Bias amplitude dependence of amplitude and phase of the longitudinal (i.e. *d*_33_) and transverse (*d*_31_) displacement with the measurement frequency of 17.777 kHz.

Having theoretically predicted the auxetic piezoelectric effect in CaBi_2_Ta_2_O_9_ of both single crystal and ceramic forms, we experimentally prepared the Na, Bi, and Ce-modified CaBi_2_Ta_2_O_9_ ceramics, where the sample synthesis is presented in the Materials and Methods section. The prepared CaBi_2_Ta_2_O_9_ ceramics show typical *Cmc*2_1_ symmetry and dense microstructure with a Curie temperature of 904°C, as shown in Fig. 2A–C. Subsequently, we characterize their coefficients *d*_33_ and *d*_31_ after poling using both direct and converse piezoelectric approaches. In the direct piezoelectric measurement approach, dynamic stress is homogeneously applied to a pair of parallel ceramic planes, on which the generated short-circuit current is simultaneously measured. To measure *d*_31_, the stress is applied along the directions perpendicular to the electrical polarization, i.e. via the side planes (Fig. 2D). For *d*_33_ measurement, the stress is applied to the planes with electrodes (Fig. S8). Details of sample preparation and piezoelectric characterization are given in Materials and Methods section. Figure 2E shows both the longitudinal (i.e. *d*_33_) and transverse (i.e. *d*_31_) piezoelectric response of the Ag/CaBi_2_Ta_2_O_9_/Ag capacitors. The amplitude of the compressive stress was normalized to 1 MPa. The current densities induced by *d*_31_ and *d*_33_ have the same phase shift of 90° with respect to the dynamic stress, indicating the same sign of *d*_31_ and *d*_33_. To quantify the values of these coefficients, we measured the current density induced by longitudinal *d*_33_ and transverse *d*_31_ as a function of the amplitude of stress, respectively. As shown in Fig. 2F, the current density increases linearly with the stress in both *d*_33_ and *d*_31_ measurement geometries. It is consistent with the formula *J*_3_ = 2*πfd*_3_*_i_σ_i_*, where *f* is the frequency of applied stress (i.e. 217.7 Hz here), *J*_3_ is the amplitude of current density, *σ_i_* is the amplitude of stress. The piezoelectric coefficient can be determined from the slope of *J*_3_ versus *σ_i_*. The experimental values of *d*_33_ and *d*_31_ in the CaBi_2_Ta_2_O_9_ ceramic are evaluated as 12.8 pC N^−1^ and 5.4 pC N^−1^, respectively. Meanwhile, the phase of *d*_33_ and *d*_31_ retains equal and constant over a wide stress range, proving its auxetic piezoelectric effect again. Frequency-dependent current density measurement under constant stress is further used to confirm the piezoelectric coefficient (Fig. S9). We also characterized the *d*_33_ and *d*_31_ of pure CaBi_2_Ta_2_O_9_ ceramic, and the values are 6.2 pC N^−1^ and 3.5 pC N^−1^ (Figs S10 and S11), respectively. These values are close to the DFT-predicted values of *d*_33_ = 8.82 pC N^−1^, *d*_31_ = 3.44 pC N^−1^. These results indicate that the intrinsic auxetic piezoelectricity remains unchanged upon doping, and the magnitude of the auxetic piezoelectric effect can be effectively tuned through simple ion doping. These dopants promote densification through heterogeneous nucleation, thereby increasing the dielectric breakdown strength, which facilitates domain reorientation during the poling process. Simultaneously, they introduce local lattice heterogeneity that perturbs long-range ferroelectric order and flattens the potential energy landscape. As a result, the piezoelectric response can be enhanced while the auxetic piezoelectricity is still preserved. For comparison, the direct piezoelectric properties of a conventional PZT-5 h ceramic were also characterized (Fig. S12). The current density induced in the longitudinal geometry (i.e. *d*_33_) exhibits 180° phase difference relative to the transverse one (i.e. *d*_31_), being consistent with the opposite signs of *d*_33_ and *d*_31_ in PZT-5 h ceramic.

To unravel the mechanical deformation of the CaBi_2_Ta_2_O_9_ ceramics under external electric field stimulus, the converse piezoelectric effect was measured. In this regard, atomic force microscope (AFM) system was used to measure the displacement of the CaBi_2_Ta_2_O_9_ ceramics under an a.c. voltage (Fig. 2G). Details of the measurement set-up and calibration methods are provided in the methods section and in Figs S13 and S14. As shown in Fig. 2H, both the longitudinal displacement (i.e. *d*_33_) and transverse displacement (i.e. *d*_31_) expand synchronously with the applied sinusoidal voltage. Their phases stay around 0°, consolidating the piezoelectric auxetic effect from the perspective of converse piezoelectric effect. To determine the converse piezoelectric coefficient, we obtained the amplitude of the displacement as a function of the bias amplitude (Fig. 2I). Based on the relation *η_i_* = *d*_3_*_i_E*_3_ where *η_i_* is the mechanical strain, *E*_3_ is the amplitude of applied electric field, the values of converse piezoelectric coefficients can be determined by the slope of *η_i_* versus *E*_3_. The converse *d*_33_ and *d*_31_ coefficients of the modified CaBi_2_Ta_2_O_9_ ceramic are 12.6 pm V^−1^ and 5.3 pm V^−1^, respectively, while those of the undoped sample decreased to 6.2 pm V^−1^ and 3.4 pm V^−1^ (Fig. S15), both in good agreement with the corresponding direct measurements. The frequency dependence of the deformation induced by the converse piezoelectric effect was also examined and showed nearly frequency-independent piezoelectric coefficients, indicating an intrinsic origin of the response (Fig. S16). In contrast, the PZT-5 h ceramic shows opposite longitudinal and transverse displacements induced by an a.c. voltage arising from the opposite signs of *d_3_*_3_ and *d*_31_ (Fig. S17). Therefore, the experimental study on the prepared CaBi_2_Ta_2_O_9_ ceramics confirms its auxetic piezoelectric nature, which is consistent with the theoretical calculation results.

The auxetic nature of the piezoelectric effect in CaBi_2_Ta_2_O_9_ ceramics can be attributed to the positive *d*_31_ and *d*_32_ of its crystal, which play an important role in altering the sign of the macroscopic *d*_31_ of the ceramics. To understand the origin of the abnormal *d*_31_ in CaBi_2_Ta_2_O_9_ crystal, we decompose the origin of *e*_31_ into the clamped-ion and internal-strain contributions, which is given as: [24]


(1) \begin{eqnarray*}
{e}_{31} = e_{31}^{(0)} + \sum\limits_s {\frac{e}{\Omega }} Z_{33,s}^*\frac{{\partial {u}_{3,s}}}{{\partial {\eta }_1}}.
\end{eqnarray*}

**Figure 3.**
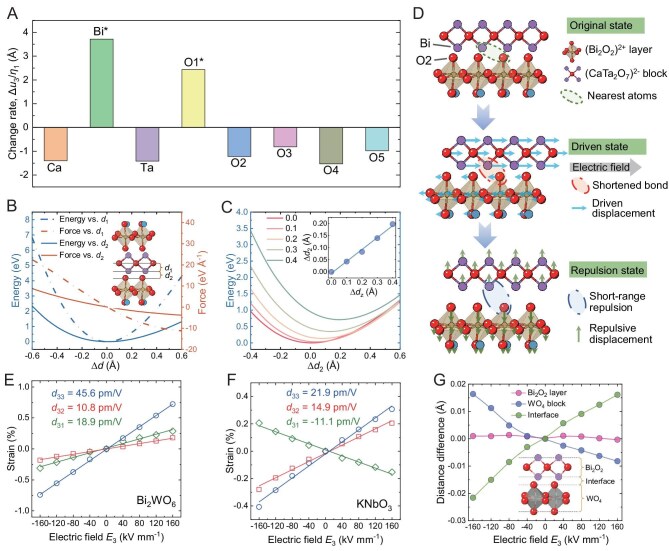
Origin of abnormal *d*_31_ in CaBi_2_Ta_2_O_9_. (A) Calculated displacement change rate of cations and anions on uniaxial strain *η*_1_, where the convention of $\mathop \sum \nolimits_i {\mathrm{\Delta }}{u}_{i{\mathrm{,3}}}{\mathrm{ = \ 0}}$ is used to eliminate the global atomic translation and keep the arithmetic center constant. * Represents the atoms in Bi_2_O_2_ layer. (B) Calculated energy and force evolution by changing the distance *d*_1_ or *d*_2_ along the *x* direction. (C) Calculated energy evolution as a function of interlayer distance change ∆*d*_2_ with various longitudinal displacements *d*_z_ of Bi_2_O_2_ layer along the +*z* direction. The inset is the dependence of ∆*d*_2_ as a function of displacement *d*_z_. (D) Schematic diagram for electric field-driven longitudinal and transverse deformation. Direct electric field calculation of (E) Bi_2_WO_6_ and (F) orthorhombic KNbO_3_. (G) Distance evolution of Bi_2_WO_6_ under external electric field, noting that a Bi_2_WO_6_ unit cell contains 2 units of Bi_2_O_2_, 2 units of WO_4_, and 4 units of interface.

The first term on the right of above the equation represents the clamped-ion contribution and its value is typically small in most ferroelectrics. The second term is the internal-strain term $e_{{\mathrm{31}}}^{{\mathrm{(}}i{\mathrm{)}}}$, arising from microscopic atomic relaxations within the lattice in response to a macroscopic transverse strain *η*_1_. Here, *s* runs over all the atoms in the unit cell of volume Ω, *e* is the electron charge, $Z_{{\mathrm{33}}}^{\mathrm{*}}$ is the Born effective charge of ferroelectric phase, and *u*_3_ is ionic displacement along the polarization direction. For CaBi_2_Ta_2_O_9_, the clamped-ion term $e_{{\mathrm{31}}}^{{\mathrm{(0)}}}$ is small and negative (−0.4 C m^−2^), whereas the internal-strain term dominates the overall positive response ($e_{{\mathrm{31}}}^{{\mathrm{(}}i{\mathrm{)}}}{\mathrm{ = 1}}{\mathrm{.57\ C\ }}{{\mathrm{m}}}^{ - {\mathrm{2}}}$). To gain a further insight into how the internal relaxation happens, we analyze the displacement change rate, ∆*u*_3_/∆*η*_1_, of each ion in response to the macroscopic uniaxial strain (Fig. 3A, Figs S18–S19, and Table S8). The displacement ∆*u*_3_ is defined as the product of internal atomic coordinate change ∆*w*_3_ and the cell parameter *c*, and the sign of change rate defines the direction of displacement change. Hence, with the application of positive *η*_1_ strain, the Bi and O1 ions in the Bi_2_O_2_ layer move to +*z* direction while the Ca, Ta, O2, O3, O4, O5 ions in the perovskite block move to −*z* direction. This suggests that a tensile transverse strain *η*_1_ induces a relative displacement between the Bi_2_O_2_ layer and perovskite block, resulting in an enhanced electrical polarization *P*_3_. Application of uniaxial stress on CaBi_2_Ta_2_O_9_ cell along other directions (i.e. *σ*_2_ and *σ*_3_) shows a similar trend of atomic displacements (Fig. S20).

To understand this, we calculated the energy and force evolution as a function of the layer distances *d*_1_ and *d*_2_, respectively, which unravels the bond strength of Bi-O (see inset of Fig. 3B). Here, *d*_1_ refers to the vertical distance between Bi and O atoms within the Bi_2_O_2_ layer, while *d*_2_ denotes the vertical distance between Bi atoms in the Bi_2_O_2_ layer and O atoms in the adjacent perovskite block. The energy difference and generated restoring force associated with *d*_1_ are approximately three times larger than that associated with *d*_2_. Precisely because of the strong anisotropy in the chemical bonding associated with the Bi atoms, the bonding within the (Bi_2_O_2_)^2+^ layer is relatively strong, whereas the interaction between the (Bi_2_O_2_)^2+^ layer and the perovskite block is really weak and flexible. Electron localization function (ELF) analysis confirms the above conclusion in Fig. S21, where the Bi and O2 show a weak ionic bond with incomplete charge transfer. As discussed below, it is this quasi-layered structure of CaBi_2_Ta_2_O_9_ lattice, along with the associated displacement between Bi_2_O_2_ layer and perovskite block, that endows positive value to *d*_31_.

To unravel this, we explore how the CaBi_2_Ta_2_O_9_ lattice deforms under an electric field using DFT calculations, especially the distance *d*_2_ between the Bi_2_O_2_ layer and perovskite block. Under a positive electric field *E*_3_, the Bi_2_O_2_ layers with nominal +2 valence charges move towards the +*z* direction whereas the perovskite blocks with −2 valence charges move towards −*z* direction, leading to longitudinal expansion (i.e. positive *d*_33_). To see how this sliding-style displacement between Bi_2_O_2_ layer and perovskite block along longitudinal direction affects their interlayer distance, we calculate the lattice energy by changing the interlayer distance ∆*d*_2_ under various longitudinal displacement *d_z_* of Bi_2_O_2_ layer along +*z* direction. Here, we set *d_z_* in the range from 0 Å to 0.4 Å with an interval of 0.1 Å. As shown in Fig. 3C, ∆*d*_2_ corresponding to the minimum energy shifts to the positive value with increasing the longitudinal sliding distance *d_z_*. Specifically, ∆*d*_2_ increases linearly with *d_z_*, confirming an expanded transverse dimension and thus positive *d*_31_ (see the inset of Fig. 3C).

Because the Bi atom is located at the center of the outer oxygen octahedron of the perovskite block, the intrinsic ferroelectric sliding causes the Bi atom to move closer to the outer oxygen atom of the perovskite block (denoted as O2) along the +*z* direction, as schematically illustrated in Fig. S22. The interlayer spacing is essentially determined by the nearest atomic pair between the Bi_2_O_2_ layer and the perovskite block, namely the Bi and O2 atoms. This implies that when an external electric field is further applied along the [001] direction, the field-driven relative displacement between the Bi_2_O_2_ layer and the perovskite block leads to a shortening of the Bi-O2 bond distance at the interface. This gives rise to a strong short-range electron Coulomb repulsion force between the Bi_2_O_2_ layer and perovskite block, leading to an increased interlayer distance *d*_2_ and transverse expansion, i.e. positive *d*_31_ (as schematically shown in Fig. 3D). Note that similar behaviors have also been discovered in other bismuth layered perovskite materials, such as Bi_2_WO_6_. We employ the direct finite electric field calculations to study the relationship between cell parameters and atomic structure of Bi_2_WO_6_ in Fig. 3E, where all strains *η*_1_, *η*_2_, and *η*_3_ increase with the electric field, showing positive *d*_31_, *d*_32_ and *d*_33_. In contrast, the orthorhombic KNbO_3_ shows negative *d*_31_ in Fig. 3F. We further study the distance evolution of internal Bi_2_WO_6_ unit cell under an external electric field in Fig. 3G and Fig. S23. The results show that the Bi_2_O_2_ layer is highly resistant to being compressed or expanded, and the thickness of the Bi_2_O_2_ layer has barely changed after applied electric field. Meanwhile, the perovskite block, WO_4_, follows the law of conventional perovskite materials, such as orthorhombic KNbO_3_ in the *x* direction, that is, the thickness of WO_4_ block decreases with the increasing electric field. Because a single Bi_2_WO_6_ unit cell contains four interfaces between the Bi_2_O_2_ layers and the WO_4_ blocks, the observed lateral expansion of Bi_2_WO_6_ can be attributed to the increased spacing between the Bi_2_O_2_ and WO_4_ units along the *x* direction, arising from electric-field-induced layer-layer lattice mismatch. This mechanism is fully consistent with our conclusions. Overall, the results highlight the critical role of relative displacement between differently charged structural units in generating a positive *d*_31_. Based on the above results, two key features can be identified as essential for realizing positive *d*_31_ in layered ferroelectrics, the presence of two distinct charged layers and spontaneous polarization oriented in the in-plane direction. Under these conditions, an external electric field applied along the polarization direction can induce relative sliding between the layers, resulting in transverse expansion. This principle provides a clear physical guideline for the design and discovery of new materials with positive *d*_31_ properties.

The positive behavior of *d*_32_ in CaBi_2_Ta_2_O_9_ crystal is similar to that in the orthorhombic KNbO_3_, where their polarization vector is along the [110] direction in the perovskite unit [25]. In this case, the B-site cation (i.e. Ta) displaces towards the edge center of oxygen octahedron. When an external electric field is applied, the displacement of B-site cation is increased to +*z* direction and that of oxygen anion is decreased to −*z* direction. Sharp narrowing of the B-O bond experiences strong short-range repulsion, which drives the two nearest oxygen atoms away from the B-site cation along the *y* direction, thereby inducing the expansion in *y* direction.

**Figure 4.**
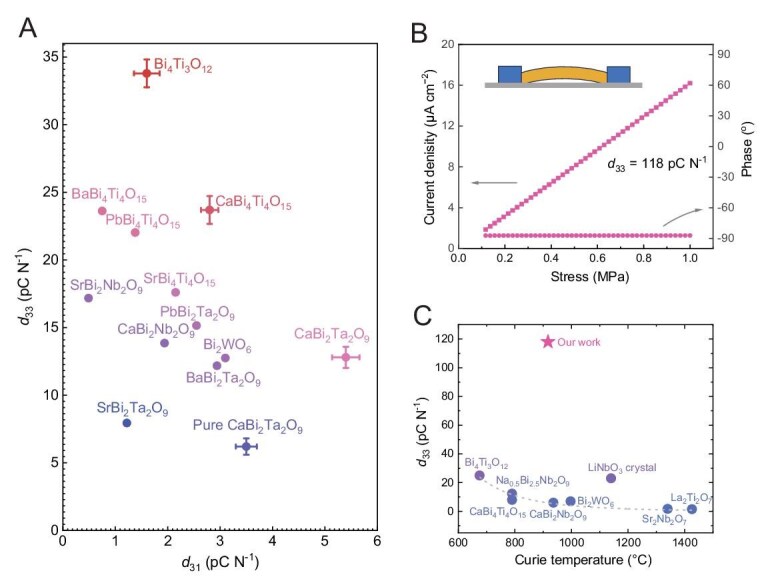
Summary of auxetic piezoelectric coefficients in Aurivillius ceramics. (A) Experimental values are represented with error bars that are statistical average of 5 samples, while calculated values are shown without error bars. (B) Stress-dependent short-circuit current density induced by piezoelectric *d*_33_ of designed curved CaBi_2_Ta_2_O_9_ ceramics with fixed transverse strain and measurement frequency of 217.7 Hz. (C) Comparison of designed curved CaBi_2_Ta_2_O_9_ ceramics with other reported high-temperature piezoceramics [26–33].

Since the charged stratified units are identified as the origin of the unique auxetic piezoelectricity auxeticity in CaBi_2_Ta_2_O_9_, the exotic behavior is supposed to be a general property in the bismuth-layered structural ferroelectric ceramics. To this end, we calculate the piezoelectric constants of various Aurivillius crystals and find that most of their ceramics exhibit auxetic piezoelectricity. To circumvent the poor density, low dielectric breakdown strength and low piezoelectric properties of pure Aurivillius ceramics, the Ce, Co, Nb-modified Bi_4_Ti_3_O_12_ and Mn, W-modified CaBi_4_Ti_4_O_15_ ceramics have also been prepared and characterized here. Results confirm their piezoelectric auxeticity (Figs S24–S27). Figure 4A and Table S9 list the calculated and experimentally confirmed ceramics with auxetic piezoelectricity. Among them, the modified CaBi_2_Ta_2_O_9_ ceramics have a maximum *d*_31_ of 5.3 pm V^−1^. Note that the ceramics always have the characteristics of easy processing and easy modification, so the higher *d*_31_ can be expected in further development.

Lastly, since *d*_31_ and *d*_33_ are of the same sign, the effective *d*_33_ can be enhanced by fixing the transverse strain in the piezoceramics. To demonstrate this scenario, we designed special spherical-shell CaBi_2_Ta_2_O_9_ ceramics to amplify the transverse stress (Figs S28–S30). The remarkably large effective *d*_33_ of 118 pC N^−1^ was achieved, nearly 10 times the original value, as shown in Fig. 4B and Figs S31–S32. The dependence of the enhanced *d*_33_ on the spherical curvature and intrinsic piezoelectric coefficients is described in the Materials and Methods section, where nearly 90% of total effective *d*_33_ is shown to originate from the amplification of transverse stress induced by the geometric structure. We also compare the obtained effective *d*_33_ with those of other high-temperature piezoceramics in Fig. 4C to provide a reference for achievable electromechanical output in practical devices. Our designed CaBi_2_Ta_2_O_9_ ceramics significantly outperform these conventional high-temperature piezoceramics, while maintaining a high Curie temperature. This result underscores the promising potential for enhancing the piezoelectric coefficient and its applications through the discovered bulk auxetic piezoelectric effect.

In summary, we have identified the bismuth layered oxides with Aurivillius structure as a catalog of bulk materials showing the auxetic piezoelectric effect. These ceramics show abnormal positive *d*_31_ and their auxetic piezoelectricity is highly robust, sustaining over a wide temperature range. The origin of transverse expansion is attributed to the strong Coulomb repulsion between Bi and O ions of different charged stratified units, which is induced by the opposite displacement between Bi_2_O_2_ layer and perovskite block along the *z* direction under external electric field. This auxetic piezoelectric effect functions in various Aurivillius ceramics. With careful design and modification, auxetic piezoelectricity with enhanced performance can be expected in various charged stratified structures. The unique characteristic allows the design of new piezoelectric applications or improvement of existing applications that are difficult to realize in conventional piezoelectric materials.

## MATERIALS AND METHODS

### Simulations methods

First-principles calculations were performed using the Vienna *ab initio* Simulation Package (VASP) based on density functional theory [34,35]. The PBEsol exchange-correlation functional and projector-augmented wave method were adopted [36,37], with a plane-wave cutoff energy of 520 eV and Γ-centered *k*-point meshes. Structural optimization, Berry-phase polarization, piezoelectric, and elastic tensors were calculated using density functional perturbation theory and finite-difference methods [38,39]. Finite electric field and uniaxial stress calculations were further conducted using the ABINIT package [40,41]. In addition, machine-learning force fields trained from *ab initio* molecular dynamics were employed to investigate temperature-dependent dynamic structures in large supercells [42,43].

### Samples preparation and characterizations

The Aurivillius ceramics were prepared using a conventional solid-state reaction technology using high-purity oxide and carbonate powders as raw materials. Four modified compositions, Ca_0.6_(Na_0.5_Bi_0.3_Ce_0.2_)_0.4_Bi_2_Ta_2_O_9_, Bi_3.97_Ce_0.03_Ti_2.99_(Co_0.5_Nb_0.5_)_0.01_O_12_, and CaBi_4_Ti_3.93_(Mn_0.5_W_0.5_)_0.07_O_15_ are chose to synthesize the samples, which are refer to nominal CaBi_2_Ta_2_O_9_, Bi_4_Ti_3_O_12_, and CaBi_4_Ti_4_O_15_ in this paper, respectively. The undoped CaBi_2_Ta_2_O_9_ ceramics were also prepared and referred to as ‘pure CaBi_2_Ta_2_O_9_’ in this paper. After ball milling, calcination, remilling, pressing, and sintering, dense ceramic pellets were obtained and electrically poled under a DC electric field in silicone oil. Phase structures were characterized by X-ray diffraction (XRD), microstructures were observed by scanning electron microscopy (SEM), and dielectric properties were measured using an impedance analyzer. For the characterization of the auxetic piezoelectric effect, direct piezoelectric coefficients (*d*_33_ and *d*_31_) were measured using a stress–charge method on carefully polished and electroded ceramic samples, where compressive stress was applied either along the polarization direction or lateral direction to collect the generated charge signals. Converse piezoelectric coefficients were characterized using a modified AFM system by detecting the electrically induced displacement under an applied AC voltage, with appropriate frequency selection and geometric correction applied for *d*_31_ measurements.

### Phenomenological theory of piezoelectricity

A standard right-handed coordinate system with proper Euler angles was employed to construct the 3 × 3 rotation matrix, which was then used to describe the anisotropic piezoelectric properties of the material along arbitrary directions. Based on the spatial distribution of the piezoelectric coefficients in Aurivillius ferroelectric crystals, an orientational averaging method was adopted to estimate the piezoelectric properties of polycrystalline ceramics [31]. The curved ceramics with a strain-constrained ring were modeled as circular Kirchhoff–Love plates with clamped edges to derive the relationship between the effective *d*_33_ and the intrinsic piezoelectric properties of the material, as well as the sample geometry. Detailed descriptions of these methods are provided in the Supplementary Materials.

## Supplementary Material

nwag295_Supplemental_File
