# Supplementary material for: Intrinsic auxetic piezoelectricity in bulk ferroelectrics
Source: Natl Sci Rev. 2026 May 20;13(12):nwag295. doi: 10.1093/nsr/nwag295 (PMC13321123; doi:10.1093/nsr/nwag295)
Supplement: nwag295_Supplemental_File [file nwag295_supplemental_file.pdf]

Supplementary Materials for

Intrinsic Auxetic Piezoelectricity in Bulk Ferroelectrics

Zhi Tan[1]†*, He-Meng Sun[2,3]†, Wei Shi[1], Shangyi Guan[1], Hui Zhang[4], Laiming Jiang[1], Qiang Chen[1], Jie Xing[1], Jianguo Zhu[1]*, Ming-Min Yang[2,3]*

[1] College of Materials Science and Engineering, Sichuan University; Chengdu, 610065, China

[2] School of Emergent Technology, the University of Science and Technology of China; Hefei, 230026, China

[3] Hefei National Laboratory; Hefei, 230088, China

[4] Shaanxi Key Laboratory of High-Orbits-Electron Materials and Protection Technology for Aerospace, School of Advanced Materials and Nanotechnology, Xidian University; Xi'an, 710126, China

* Corresponding Author. Zhi Tan, E-mail: tanzhi0838@scu.edu.cn; Jianguo Zhu, E-mail: nic0400@scu.edu.cn; Ming-min Yang, E-mail: mingminyang@hfnl.cn

† These authors contributed equally to this work.

The PDF file includes:

Detailed materials and methods

Figs. S1 to S32

Tables S1 to S9

Contents:

Section S1: Detailed materials and methods

First-principle calculations

Molecular dynamics simulations

Sample preparation

Structural characterization

Orientation dependence of piezoelectric constants

Orientational average method

Characterization of the auxetic piezoelectric effect

Effective piezoelectric coefficient d_{33} of curved ceramics with fixed transverse strain

Section S2: Calculated elastic and piezoelectric constants of bismuth-layered ferroelectric.

Table S1. Cell parameters of calculated bismuth-layered ferroelectrics.

Table S2. Elastic stiffness constants of calculated bismuth-layered ferroelectrics.

Table S3. Piezoelectric stress constants of orthorhombic bismuth-layered ferroelectrics.

Table S4. Piezoelectric strain constants of orthorhombic bismuth-layered ferroelectrics.

Fig. S1. Dependence of (A) polarization P_3 and (B) energy on uniaxial strain using SCAN meta-GGA functional.

Section S3: Crystallographic-orientation-dependent piezoelectric constants in $CaBi_2Ta_2O_9$.

Fig. S2. Used Euler angles geometrical definition.

Fig. S3. Crystallographic-orientation-dependent piezoelectric constants in $CaBi_2Ta_2O_9$.

Fig. S4. Angle (θ)-dependent piezoelectric coefficients d_{31}, d_{32} and d_{33} in $CaBi_2TaO_9$ crystal.

Fig. S5. Schematic illustration of domain orientation in a piezoelectric ceramic disk before and after poling.

Section S4: Calculated piezoelectric constants of bismuth-layered structure ceramics.

Table S5. Predicted piezoelectric strain constants of orthorhombic bismuth-layered structure ceramics.

Section S5: Predicted piezoelectric properties at finite temperature.

Fig. S6. Polarization and dielectric constants of $CaBi_2Ta_2O_9$ at finite temperature.

Fig. S7. Calculated temperature-dependent piezoelectric constant for $CaBi_2Ta_2O_9$ ceramics.

Table S6. Calculated piezoelectric strain constants of $CaBi_2Ta_2O_9$ crystal at finite temperature.

Table S7. Calculated piezoelectric strain constants of $CaBi_2Ta_2O_9$ ceramics.

Section S6: Piezoelectric characterization of $CaBi_2Ta_2O_9$ and PZT ceramics

Fig. S8. Schematics showing the measurement geometry to characterize direct piezoelectric coefficients.

Fig. S9. Frequency dependent direct piezoelectric response of modified $CaBi_2Ta_2O_9$ ceramic.

Fig. S10. Direct piezoelectric characterization of pure $CaBi_2Ta_2O_9$ ceramics with piezoelectric auxeticity.

Fig. S11. Frequency dependent direct piezoelectric response of pure $CaBi_2Ta_2O_9$ ceramic.

Fig. S12. Direct piezoelectric characterization of PZT-5h ceramic with conventional piezoelectric effect.

Fig. S13. Schematics showing the measurement geometry to characterize converse piezoelectric coefficients.

Fig. S14. Converse piezoelectric characterization of X-cut quartz single crystal using AFM system.

Fig. S15. Converse piezoelectric characterization of pure $CaBi_2Ta_2O_9$ ceramics with piezoelectric auxeticity.

Fig. S16. Frequency-dependent converse piezoelectric response of modified $CaBi_2Ta_2O_9$ ceramics.

Fig. S17. Converse piezoelectric characterization of PZT-5h ceramics with conventional piezoelectric effect.

Section S7: Mechanism of abnormal d_{31} in $CaBi_2Ta_2O_9$.

Table S8. Calculated structural parameters of orthorhombic $CaBi_2Ta_2O_9$.

Fig. S18. Crystal structure diagram with atom labels.

Fig. S19. Displacements Δu_i of different atoms in $CaBi_2Ta_2O_9$ under the uniaxial strain.

Fig. S20. Displacements Δu_i of different atoms in $CaBi_2Ta_2O_9$ under the uniaxial stress.

Fig. S21. Electron localization function (ELF) of $CaBi_2Ta_2O_9$.

Fig. S22. Schematic diagrams of interface between Bi_2O_2 layer and perovskite block.

Section S8: Mechanism of abnormal d_{31} in Bi_2WO_6.

Fig. S23. Weighted distance evolution of Bi_2WO_6 under external electric field.

Section S9: Piezoelectric characterization of other bismuth-layered structure ceramics.

Fig. S24. Direct piezoelectric characterization of $CaBi_4Ti_4O_{15}$ ceramics with auxetic piezoelectric effect.

Fig. S25. Converse piezoelectric characterization of $CaBi_4Ti_4O_{15}$ ceramics with auxetic piezoelectric effect.

Fig. S26. Direct piezoelectric characterization of $Bi_4Ti_3O_{12}$ ceramics with auxetic piezoelectric effect.

Fig. S27. Converse piezoelectric characterization of $Bi_4Ti_3O_{12}$ ceramics with auxetic piezoelectric effect.

Table S9. Experimentally measured Curie temperatures and piezoelectric coefficients together with their uncertainties for the investigated materials.

Section S10: Characterization of clamped piezoelectric ceramics.

Fig. S28. Schematic diagram of designed $CaBi_2Nb_2O_9$ ceramics with fixed transverse strain.

Fig. S29. Illustration of the deformation of the curved ceramic under an applied external force.

Fig. S30. Curvature characterization of designed $CaBi_2Nb_2O_9$ ceramics.

Fig. S31. Direct piezoelectric characterization of clamped $CaBi_2Nb_2O_9$ ceramic.

Fig. S32. Converse piezoelectric characterization of clamped $CaBi_2Nb_2O_9$ ceramic.

Section S1: Detailed materials and methods

First-principle calculations

The first-principle calculations in the present work are carried out using Vienna Ab initio Simulation Package (VASP) based on density functional theory (DFT) [S1,S2]. The PBEsol [S3] exchange-correlation functional with projector-augmented wave (PAW) [S4] is employed in the calculations. The Ca $3s^23p^64s^2$, Bi $5d^{10}6s^26p^3$, Ta $5p^66s^25d^3$, La $5s^25p^66s^25d^1$, Ti $3s^23p^64s^23d^2$, and O $2s^22p^4$ states are treated as valence electrons for calculations. The wave function is represented as a plane wave expansion that is truncated at a cut-off energy of 520 eV. Γ-centered k-point meshes with a grid of spacing $0.04 \times 2\pi$ Å^{-1} for Brillouin zone sampling were chosen. The Kohn-Sham orbitals are updated in the self-consistency cycle until an energy convergence of 10^{-6} eV is obtained, and the geometry optimizations are completed when the residual force of each atom is less than 0.002 eV Å^{-1}. The macroscopic polarization is defined by the so-called modern theory of polarization using the Berry phase method [S5]. The piezoelectric and elastic tensors as the second derivatives of total internal energy are extracted by density functional perturbation theory (DFPT) [S6] and finite differences method. Finite electric field [S7] and uniaxial stress calculations are performed using the ABINIT package [S8] based on WC exchange-correlation functional [S9] with PAW.

Molecular dynamics simulations

The on-the-fly machine learning force fields (MLFF) [S10,S11] are trained in a 1 × 2 × 2 supercell of $Cmc2_1$ $CaBi_2Ta_2O_9$ structure contained 224 atoms to conduct the ab initio molecular dynamics (AIMD) based on VASP. In the training process, the simulations are executed in the *NPT* ensemble for 20 ps using the time step of 1 fs with energy convergence criterion of 10^{-4} eV at 5, 100, 300, 500, 800, 1000, and 1200 K, respectively. Total 985 configurations are collected in the training data set. After collection completion, we conduct the refitting of the machine learning force field on a larger threshold with value of 1×10^{-8} to smooth the force field for speeding up subsequent simulations. The root mean squared errors (RMSEs) of energies, forces, and stress of obtained MLFF predictions with respect to ab initio results for the training data are 8.458×10^{-4} eV per atom, 0.1261 eV Å^{-1}, and 0.9632 kB, respectively. Based on the generated MLFF, the 2 × 8 × 8 supercell contained 7168 atoms with total MD simulations times of 100 ps are used to obtain the dynamic structures at constant temperatures of 5, 100, 300, 500, 600, 700, 800, 900, 1000, 1100 K, respectively. The piezoelectric strain constants d_{ij} at finite temperature are calculated by

$$d_{ij} = \frac{1}{k_B T}\left\langle \Delta M_i \Delta \eta_j \right\rangle . \qquad \text{(S1)}$$

Here, k_B is the Boltzmann constant, T is the temperature, and η is the strain tensor, in which η is obtained by

$$\eta = \frac{1}{2}\left(H_0^{t^{-1}} G H_0^{-1} - 1 \right), \qquad \text{(S2)}$$

where $G = H^tH$ with $H = \{a, b, c\}$ representing the MD cell, and H_0 is the reference state of H. M_k is the kth total dipole moment of supercell, and $\Delta X = X - \langle X \rangle$.

Sample preparation

The Aurivillius ceramics were prepared using a conventional solid-state reaction technology. Four modified compositions, $Ca_{0.6}(Na_{0.5}Bi_{0.3}Ce_{0.2})_{0.4}Bi_2Ta_2O_9$, $Bi_{3.97}Ce_{0.03}Ti_{2.99}(Co_{0.5}Nb_{0.5})_{0.01}O_{12}$, and $CaBi_4Ti_{3.93}(Mn_{0.5}W_{0.5})_{0.07}O_{15}$ are chose to synthesize the samples, which are refer to nominal $CaBi_2Ta_2O_9$, $Bi_4Ti_3O_{12}$, and $CaBi_4Ti_4O_{15}$ in this paper, respectively(*45, 46*). The undoped $CaBi_2Ta_2O_9$ ceramics were also prepared and referred to as ‘pure $CaBi_2Ta_2O_9$’ in this paper. $CaCO_3$ (99.0%), Na_2CO_3 (99.5%), Bi_2O_3 (99.999%), CeO_2

(99.999%), TiO_2 (98.0%), Ta_2O_5 (99.999%), Nb_2O_5 (99.5%), Co_2O_3 (99%), and WO_3 (99%) powders were used as the starting raw materials and weighed according to the stoichiometric compositions. After being mixed through planetary ball mill with ethanol for 24 h, the mixture was calcined at 900 ℃ for 3 h. Then continued mixed in a total time of 24 h was conducted to ensure the uniformity. After drying, the powders were pressed into pellets using the binder of 8 wt% polyvinyl alcohol (PVA). Finally, the samples with the PVA removed were sintered in air at 1200, 1150, and 1000 ℃ for $CaBi_2Ta_2O_9$, $Bi_4Ti_3O_{12}$, and $CaBi_4Ti_4O_{15}$, respectively. The top and bottom surfaces of the specimens are printed with silver paste and then fired at 700–750 °C for 10 mins to form dense electrodes. These obtained pellets were poled in silicon oil under a DC electric field of 8–12 kV mm^{-1} for 20 mins at 200 °C.

Structural characterization

X-ray diffraction (XRD, X'Pert Pro MPD, B.V. PANalytical) with Cu Ka radiation was used to measure the phase structure of all samples. The dielectric constant and dielectric loss as a function of temperature are determined using the precision impedance analyzer (TH2827, Tonghui Electronic Co, China). The surface morphologies were characterized by a scanning electron microscopy (SEM, S-3400N, Hitachi).

Orientation dependence of piezoelectric constants

The anisotropy of the Aurivillius crystal leads to the anisotropic physical properties, and these physical properties at arbitrary direction are a function of spatial direction. The calculations of the physical properties as a function of orientation can be done via coordinate transforms. Here, a standard right-hand set with proper Euler angles is used to define the spatial orientation. The piezoelectric constants at arbitrary direction can be calculated via the 3×3 rotation matrix:

$$\boldsymbol{R}(\psi,\ \theta,\ \varphi)=\begin{pmatrix} a_{11} & a_{12} & a_{13} \\ a_{21} & a_{22} & a_{23} \\ a_{31} & a_{32} & a_{33} \end{pmatrix}, \tag{S3}$$

where a_{ij} is the transformation matrix elements and defined as the cosines of angles between the original axis x_i^0 in the Cartesian coordinate system $\{x_1^0,\ x_2^0,\ x_3^0\}$ and the new axis x_j' in $\{x_1',\ x_2',\ x_3'\}$ (see Fig. S1). The a_{ij} at here is written in term of the Euler angles (ψ, θ, φ) in the *ZXZ* convention, which describes the continuous and anticlockwise rotations in the ordinate system about the x_3^0 axis (by ψ), x_1^0 axis (by θ), and the final x_3' (by φ). In this case, the elements a_{ij} are written as:

$$\begin{aligned} a_{11} &= \cos\varphi\cos\psi-\cos\theta\sin\psi\sin\varphi, \\ a_{12} &= \cos\varphi\sin\psi+\cos\theta\cos\psi\sin\varphi, \\ a_{13} &= \sin\varphi\sin\theta, \\ a_{21} &= -\sin\varphi\cos\psi-\cos\theta\sin\psi\cos\varphi, \\ a_{22} &= -\sin\varphi\sin\psi+\cos\theta\cos\psi\cos\varphi, \\ a_{23} &= \cos\varphi\sin\theta, \\ a_{31} &= \sin\theta\sin\psi, \\ a_{32} &= -\sin\theta\cos\psi, \\ a_{33} &= \cos\theta. \end{aligned} \tag{S4}$$

Each piezoelectric constant at arbitrary direction can be written as a linear transformation of original piezoelectric matrix in tensor notation:

$$d'_{ijk} = a_{im}a_{jn}a_{kp}d^0_{ijk}\ , \tag{S5}$$

where the *i*, *j*, *k*, *m*, *n*, *p* = {*x*, *y*, *z*} in Cartesian directions, *x* ~ 1, *y* ~ 2, and *z* ~ 3, full notation and the Einstein summation is used here. The piezoelectric constants in Voigt notation are related to the full tensor components as follows, $d_{31} = d_{311}$, $d_{32} = d_{322}$, $d_{33} = d_{333}$, $d_{24} = 2d_{223}$, d_{15}

= $2d_{113}$.

Orientational average method

We use the orientational average method to estimate the piezoelectric constants of polycrystal ceramics. It should be noted that the conventional piezoelectric ceramics is an assemble of numerous small grains with disorderly random orientation. For the $CaBi_2Ta_2O_9$ ferroelectrics with $Cmc2_1$ space group, the polarization can be reorientated in four possible equivalent directions, [010], [0$\bar{1}$0], [001], and [00$\bar{1}$]. Supposing domain switching is completed during the poling process, the polarization of each grain should be induced in a nearest possible direction following the external electric field. Therefore, an orientational average in the region closest to the polarization axis can represent the piezoelectric characteristics of conventional $CaBi_2Ta_2O_9$ ceramics. Due to the symmetry and the boundary condition, the piezoelectric constants of poled ceramics can be evaluated by [S12]

$$d_{ijk,ceramics} = \frac{\int_0^{\pi/2}\int_0^{\mathrm{arccot}(\cos\psi)}\int_0^{2\pi} d'_{ijk}\sin\theta d\psi d\theta d\varphi}{\int_0^{\pi/2}\int_0^{\mathrm{arccot}(\cos\psi)}\int_0^{2\pi}\sin\theta d\psi d\theta d\varphi}. \tag{S6}$$

After calculation, we can find the relation between the piezoelectric constant of poled ceramics and these of the single crystal. Only three independent piezoelectric constants exist in poled piezoelectric ceramics, and the piezoelectric constants of Aurivillius ceramics can be written as

$$d_{33,ceramics} = \frac{\sqrt{2}}{8}(d_{31}+d_{15}) + \frac{\sqrt{2}}{16}(d_{32}+d_{24}) + \frac{5\sqrt{2}}{16}d_{33}, \tag{S7}$$

$$d_{31,ceramics} = \frac{3\sqrt{2}}{16}d_{31} + \frac{7\sqrt{2}}{32}d_{32} + \frac{3\sqrt{2}}{32}d_{33} - \frac{\sqrt{2}}{32}d_{24} - \frac{\sqrt{2}}{16}d_{15}, \tag{S8}$$

$$d_{15,ceramics} = -\frac{\sqrt{2}}{16}d_{31} - \frac{\sqrt{2}}{32}d_{32} + \frac{3\sqrt{2}}{32}d_{33} + \frac{7\sqrt{2}}{32}d_{24} + \frac{3\sqrt{2}}{16}d_{15}. \tag{S9}$$

Characterization of the auxetic piezoelectric effect

The dimensions of the ceramic samples were determined to be 3 mm × 2.2 mm× ~0.6 mm using blade saws and home-build polishing tools. The top and bottom surfaces were polished flat for subsequent electrode preparation, and the side edges were polished to be parallel to minimize inhomogeneous stress. Before sputtering silver electrode (ET-NanoSputter from Anhui Epitaxy Technology Co. Ltd), the ceramic surfaces were subjected to ultrasonic cleaning with alcoholic for 1 minute and then cleaned with oxygen plasma for 1 minute.

Supplementary Fig. S6 shows the schematics of direct d_{33} and d_{31} measurement setups. To measure d_{33} of the flat (parallel-plate) piezoelectric ceramics, the Ag/ceramic/Ag capacitors were clamped between two conducting substrates, that is $La_{0.67}Ca_{0.33}MnO_3$ conductive ceramics or sapphire substrates coated with gold electrodes in our experiment. To measure d_{31}, the stress is applied to the parallel lateral edges of the sample using two parallel insulated sapphire substrates. For the curved (arch-shaped) ceramics, the effective d_{33} was measured using the same stress-charge configuration. The bottom electrode of the curved ceramic was electrically connected to the clamped stainless-steel plate, while the top electrode was directly contacted by a conductive block, as in the measurement of the flat ceramic, so that the generated charge signal could be collected under mechanical loading. When mounting the samples for d_{33} and d_{31} measurement, care must be taken to ensure that the polarization direction is consistently oriented towards the input terminals of the lock-in amplifier or oscilloscope. This is important for the uniformity of the signs of d_{33} and d_{31}.

To detect the converse piezoelectric effect, a modified AFM system was used to detect the deformation of the piezoelectric ceramics under applied voltage. Similarly to direct

piezoelectric measurements, it is crucial to ensure that, in the d_{33} and d_{31} measurement, the direction of applied voltage should be consistently aligned with the polarization direction of piezoelectric ceramics. Before measuring the piezoelectric coefficient, the deflection inverse optical lever sensitivity (Delf InOLS) should be determined from the force-distance curve. To accurately measure the displacement, it is crucial to ensure that the probe and the top surface of the sample are at the same electric potential to minimize the influence of electrostatic effects. We usually chose the frequency of 17.777 kHz for the driven bias. The frequency below a certain threshold (e.g., 500 Hz) are susceptible to environmental noise, while higher frequencies near the resonance frequency of probe cantilever (around 260 kHz) can significantly affect the value of displacement. Besides, in the d_{31} measurement, the displacement needs to be normalized by dividing by the coefficient of h/t, where h is the length of the sample in the displacement direction, and t is the thickness of sample in the applied electric field direction. In the d_{33} measurement, h/t is equal to 1. For the curved (arch-shaped) ceramics, the converse piezoelectric response was measured using the same AFM-based approach, with an AC voltage applied between the top and bottom electrodes and the resulting out-of-plane displacement detected.

Effective piezoelectric coefficient d_{33} of curved ceramics with fixed transverse strain

We prepared a curved ceramics with fixed strain to demonstrate the enhances of d_{33} [S13]. The curved ceramics with strain-bound ring can be simply viewed as a circular Kirchhoff-Love plates with clamped edges [S14]. We have deflection w and slope of the deflection surface ϕ are zero at the edge of the plate (radius a). In cylindrical coordinates these boundary conditions can obtain

$$w(r) = -\frac{q}{64D}(a^2 - r^2)^2 , \tag{S10}$$

$$\phi(r) = -\frac{qr}{16D}(a^2 - r^2) , \tag{S11}$$

where q is the applied load per unit area and the D is the bending stiffness, and in a plate with thickness of $2t$, it has

$$D = \frac{2Et^3}{3(1-v^2)} , \tag{S12}$$

where E is the Young's modulus and v is the Poisson's ratio. The in-plane displacement is

$$u_r(r) = -z\phi(r) . \tag{S13}$$

Hence, the in-plane strains in the plate are

$$\eta_{rr} = \frac{du_r}{dr} = -\frac{qz}{16D}(a^2 - 3r^2) , \tag{S14}$$

$$\eta_{\theta\theta} = \frac{u_r}{r} = -\frac{qz}{16D}(a^2 - r^2) , \tag{S15}$$

Then the in-plane stress is

$$\sigma_{rr} = \frac{E}{1-v^2}(\eta_{rr} + v\eta_{\theta\theta}) = -\frac{3qz}{32t^3}[(1+v)a^2 - (3+v)r^2] , \tag{S16}$$

We consider that the force F_z is acts on the center of curved ceramics. The maximum deflection at the center is [S15]

$$w(0) = -\frac{F_z a^2}{16\pi D} . \tag{S17}$$

Since our ceramic itself has a certain deflection, the load roughly equivalent to

$$q \approx \frac{4F_z \cos(a\rho)}{\pi a^2}, \tag{S18}$$

where ρ are curvature of ceramics. Only the extension part offers force, hence, the total transverse force of ceramics at $r = a$ can be obtained

$$F_{rr} = \int_0^t 2\pi a \sigma_{aa} dz = \frac{3a \cos(a\rho)}{2t} F_z . \tag{S19}$$

We then can estimate the total effective longitudinal piezoelectric constant as

$$d_{33,eff} = \frac{3a \cos(a\rho)}{2t} d_{31} + d_{33} . \tag{S20}$$

In our case of $CaBi_2Ta_2O_9$ ceramics, a = 5.5 mm, t = 0.33 mm, and ρ = 0.05 mm^{-1}, we can obtain

$$d_{33,eff} = 24d_{31} + d_{33} , \tag{S21}$$

which is basically consistent with our results.

Reference

S1. Kresse G, Furthmüller J. Efficient iterative schemes for *ab initio* total-energy calculations using a plane-wave basis set. *Phys Rev B* 1996; **54**: 11169–86.

S2. Kresse G, Hafner J. *Ab initio* molecular-dynamics simulation of the liquid-metal-amorphous-semiconductor transition in germanium. *Phys Rev B* 1994; **49**: 14251–69.

S3. Perdew JP, Ruzsinszky A, Csonka GI, et al. Restoring the density-gradient expansion for exchange in solids and surfaces. *Phys Rev Lett* 2008; **100**: 136406.

S4. Blöchl PE. Projector augmented-wave method. *Phys Rev B* 1994; **50**: 17953–79.

S5. King-Smith R D, Vanderbilt D. Theory of polarization of crystalline solids. *Phys Rev B* 1993; **47**: 1651–4.

S6. Wu X, Vanderbilt D, Hamann D R. Systematic treatment of displacements, strains, and electric fields in density-functional perturbation theory. *Phys Rev B* 2005; **72**: 035105.

S7. Stengel M, Spaldin NA, Vanderbilt D. Electric displacement as the fundamental variable in electronic-structure calculations. *Nat Phys* 2009; **5**: 304–8.

S8. Torrent M, Jollet F, Bottin F, et al. Implementation of the projector augmented-wave method in the ABINIT code: Application to the study of iron under pressure. *Comput Mater Sci* 2008; **42**: 337–51.

S9. Wu Z, Cohen R E. More accurate generalized gradient approximation for solids. *Phys Rev B* 2006; **73**: 235116.

S10. Jinnouchi R, Karsai F, Kresse G. On-the-fly machine learning force field generation: application to melting points. *Phys Rev B* 2019; **100**: 014105.

S11. Jinnouchi R, Lahnsteiner J, Karsai F, et al. Phase transitions of hybrid perovskites simulated by machine-learning force fields trained on the fly with Bayesian inference. *Phys Rev Lett* 2019; **122**: 225701.

S12. Tan Z, Xi J, Xing J, et al. Understanding the piezoelectric origin of bismuth layer-structured ferroelectric polycrystal using first-principle method. *J Eur Ceram Soc* 2022; **42**: 3865–76.

S13. Tan Z, Zhu J, Yang MM, et al. A kind of high-performance bismuth-layer structure piezoelectric ceramics and their preparation method. *Chinese Patent* 2025; **Application No. 202510061540.9**.

S14. Timoshenko S, Woinowsky-Krieger S. *Theory of Plates and Shells*. New York: McGraw-Hill, 1959.

S15. Young WC, Budynas RG. *Roark's Formulas for Stress and Strain*. New York: McGraw-hill, 2002.

Section S2: Calculated elastic and piezoelectric constants based of bismuth-layered ferroelectrics.

Table S1. Cell parameters of calculated orthorhombic bismuth-layered ferroelectrics.

Materials	Space group	*a* (Å)	*b* (Å)	*c* (Å)	Volume (Å^3)
Bi_2WO_6	*Bba*2	16.538	5.467	5.469	494.72
$CaBi_2Nb_2O_9$	$Cmc2_1$	24.775	5.434	5.494	739.55
$SrBi_2Nb_2O_9$	$Cmc2_1$	24.846	5.541	5.546	763.51
$BaBi_2Nb_2O_9$	$Cmc2_1$	24.939	5.601	5.617	784.47
$CaBi_2Ta_2O_9$	$Cmc2_1$	24.985	5.424	5.473	741.78
$SrBi_2Ta_2O_9$	$Cmc2_1$	25.027	5.540	5.540	768.18
$BaBi_2Ta_2O_9$	$Cmc2_1$	25.105	5.589	5.604	786.36
$PbBi_2Ta_2O_9$	$Cmc2_1$	25.063	5.552	5.559	773.56
$Bi_4Ti_3O_{12}$	*Bba*2	32.762	5.407	5.438	963.32
$CaBi_4Ti_4O_{15}$	$Cmc2_1$	40.561	5.400	5.428	1188.79
$SrBi_4Ti_4O_{15}$	$Cmc2_1$	40.475	5.445	5.470	1205.43
$BaBi_4Ti_4O_{15}$	$Cmc2_1$	40.438	5.486	5.531	1227.12
$PbBi_4Ti_4O_{15}$	$Cmc2_1$	40.462	5.461	5.490	1213.23

Table S2. Elastic stiffness constants of calculated orthorhombic bismuth-layered ferroelectrics, in the unit of GPa.

Materials	C_{11}	C_{12}	C_{13}	C_{22}	C_{23}	C_{33}	C_{44}	C_{55}	C_{66}
Bi_2WO_6	122.1	66.4	32.9	202.1	54.8	175.9	55.8	56.4	76.1
$CaBi_2Nb_2O_9$	149.0	57.8	33.2	204.6	41.0	173.0	54.4	57.0	73.4
$SrBi_2Nb_2O_9$	136.2	52.1	32.3	200.2	37.9	180.6	54.4	59.7	64.7
$BaBi_2Nb_2O_9$	120.0	49.0	30.9	227.4	44.8	194.4	50.3	61.0	46.0
$CaBi_2Ta_2O_9$	155.0	50.9	23.9	215.4	50.0	191.0	88.2	53.9	76.1
$SrBi_2Ta_2O_9$	136.9	44.8	22.8	213.9	50.7	207.5	97.9	59.1	67.1
$BaBi_2Ta_2O_9$	124.1	46.2	27.4	266.3	78.8	240.5	108.9	62.2	55.1
$PbBi_2Ta_2O_9$	132.8	50.5	27.4	190.4	34.4	177.0	80.8	58.0	64.4
$Bi_4Ti_3O_{12}$	143.6	78.1	50.0	231.8	44.3	182.0	57.6	57.7	73.8
$CaBi_4Ti_4O_{15}$	147.9	80.1	53.1	236.8	43.9	192.2	57.9	63.2	77.8
$SrBi_4Ti_4O_{15}$	157.2	80.1	52.8	241.3	41.9	188.1	51.1	66.5	77.7
$BaBi_4Ti_4O_{15}$	155.2	68.7	44.9	240.5	32.4	185.5	40.0	67.9	74.8
$PbBi_4Ti_4O_{15}$	148.8	80.7	55.4	235.0	38.3	174.3	43.2	65.3	74.6

Table S3. Piezoelectric stress constants of orthorhombic bismuth-layered ferroelectrics, in the unit of C m^{-2}.

Materials	e_{31}	e_{32}	e_{33}	e_{24}	e_{15}
Bi_2WO_6	1.23	2.74	3.59	2.86	-0.14
$CaBi_2Nb_2O_9$	0.97	2.14	3.47	3.46	-0.15
$SrBi_2Nb_2O_9$	0.93	2.05	3.74	4.39	0.33
$BaBi_2Nb_2O_9$	1.30	3.84	5.00	5.70	1.81
$CaBi_2Ta_2O_9$	1.28	1.59	2.98	2.68	-0.40
$SrBi_2Ta_2O_9$	1.16	0.26	2.02	3.04	-0.06
$BaBi_2Ta_2O_9$	1.50	2.74	3.75	3.32	0.69
$PbBi_2Ta_2O_9$	1.44	1.78	3.64	4.35	0.24
$Bi_4Ti_3O_{12}$	2.25	3.39	4.70	3.22	-0.27
$CaBi_4Ti_4O_{15}$	2.18	2.98	4.46	3.28	-0.03
$SrBi_4Ti_4O_{15}$	2.11	2.96	4.34	3.96	0.18
$BaBi_4Ti_4O_{15}$	2.00	3.43	4.75	5.19	0.24
$PbBi_4Ti_4O_{15}$	2.03	3.30	4.93	4.73	0.00

Table S4. Piezoelectric strain constants of orthorhombic bismuth-layered ferroelectrics, in the unit of pC N^{-1}.

Materials	d_{31}	d_{32}	d_{33}	d_{24}	d_{15}
Bi_2WO_6	0.63	8.56	17.61	51.19	-2.40
$CaBi_2Nb_2O_9$	-0.23	6.79	18.50	63.16	-2.64
$SrBi_2Nb_2O_9$	-0.28	6.66	19.32	80.72	5.47
$BaBi_2Nb_2O_9$	-0.08	12.39	22.85	113.36	29.68
$CaBi_2Ta_2O_9$	5.11	2.86	14.24	30.33	-7.42
$SrBi_2Ta_2O_9$	7.78	-2.67	9.57	31.03	-0.95
$BaBi_2Ta_2O_9$	7.31	5.15	13.08	30.49	11.07
$PbBi_2Ta_2O_9$	5.22	4.58	18.84	54.00	4.08
$Bi_4Ti_3O_{12}$	2.68	9.35	22.80	56.05	-4.74
$CaBi_4Ti_4O_{15}$	3.47	7.74	19.77	56.81	-0.53
$SrBi_4Ti_4O_{15}$	2.51	7.82	20.64	77.64	2.68
$BaBi_4Ti_4O_{15}$	1.38	10.74	23.40	129.79	3.49
$PbBi_4Ti_4O_{15}$	-1.91	10.35	26.64	109.58	-0.01

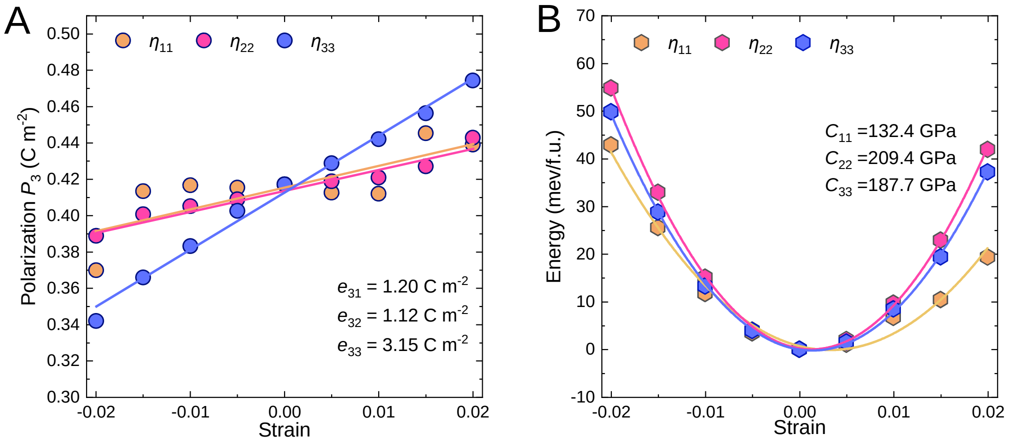

Fig. S1. Dependence of (A) polarization P_3 and (B) energy on uniaxial strain using SCAN meta-GGA functional. The SCAN functional is one of the most accurate functionals for capturing ferroelectric distortions, and the calculated results are consistent with those obtained using the PBEsol GGA functional.

Section S3: Crystallographic-orientation-dependent piezoelectric constants in $CaBi_2Ta_2O_9$.

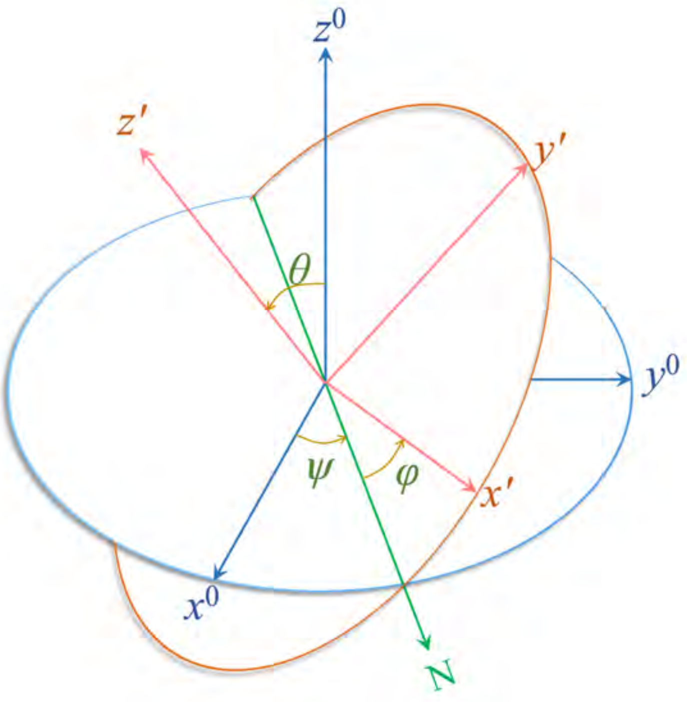

Fig. S2. Used Euler angles geometrical definition. Schematic showing the geometrical correlation between new coordinate set {x', y', z'} and default set {x^0, y^0, z^0}.

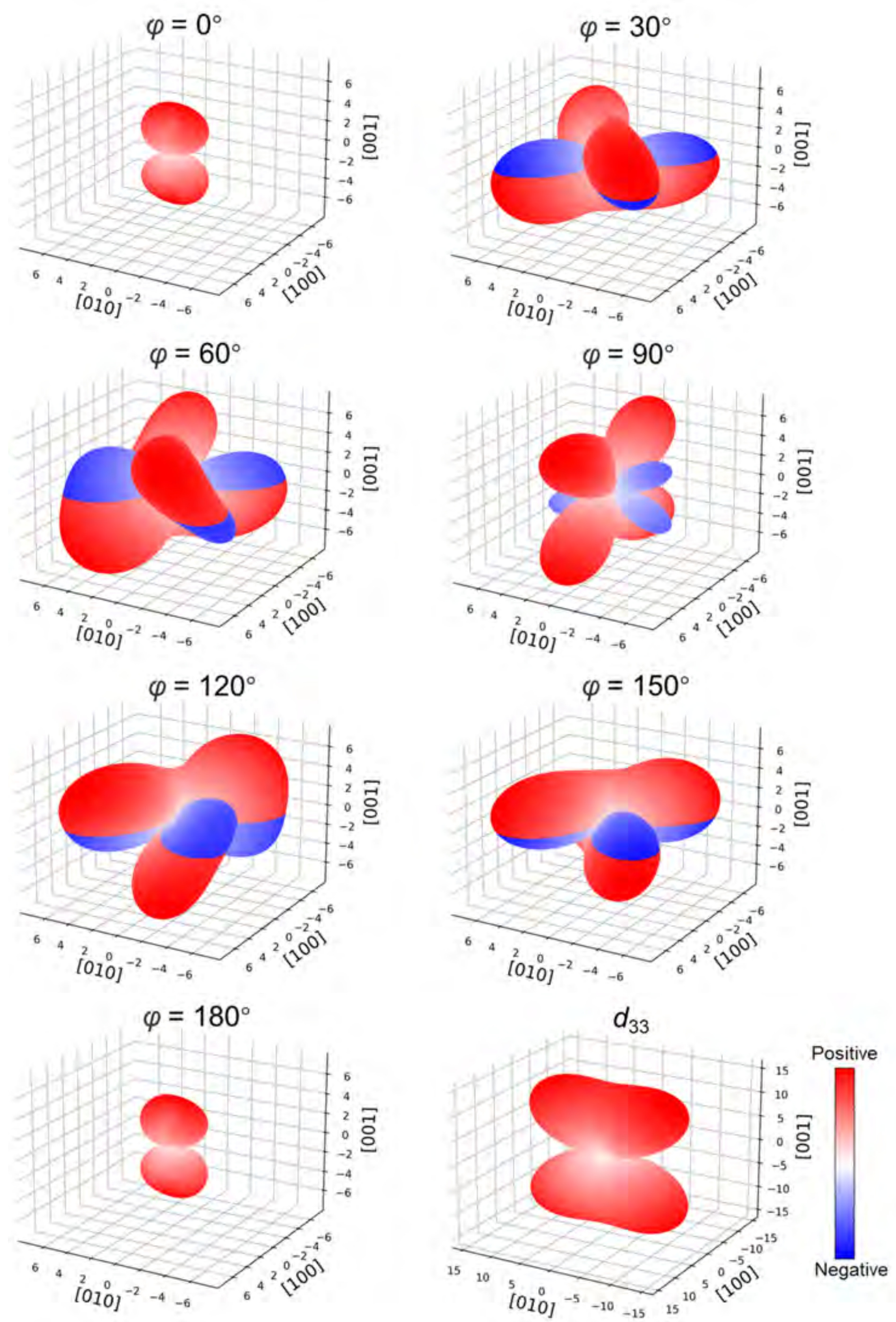

Fig. S3. Crystallographic-orientation-dependent piezoelectric constants in $CaBi_2Ta_2O_9$. The three-dimensional spherical polar plots of the crystallographic-orientation-dependent transverse piezoelectric constant d_{31} at different φ level and longitudinal piezoelectric coefficient d_{33} in $CaBi_2Ta_2O_9$ crystal. It should be noted that the φ is the rotation angle around the z-axis in Euler system, and the phase difference between d_{31} and d_{32} is 90°, which means the d_{31} at φ = 90° is equal to the d_{32} at φ = 0°. The d_{31} shows positive value in the most cases. It should be noted that d_{33} is independent of φ, because varying φ does not alter the orientation of the z'-axis.

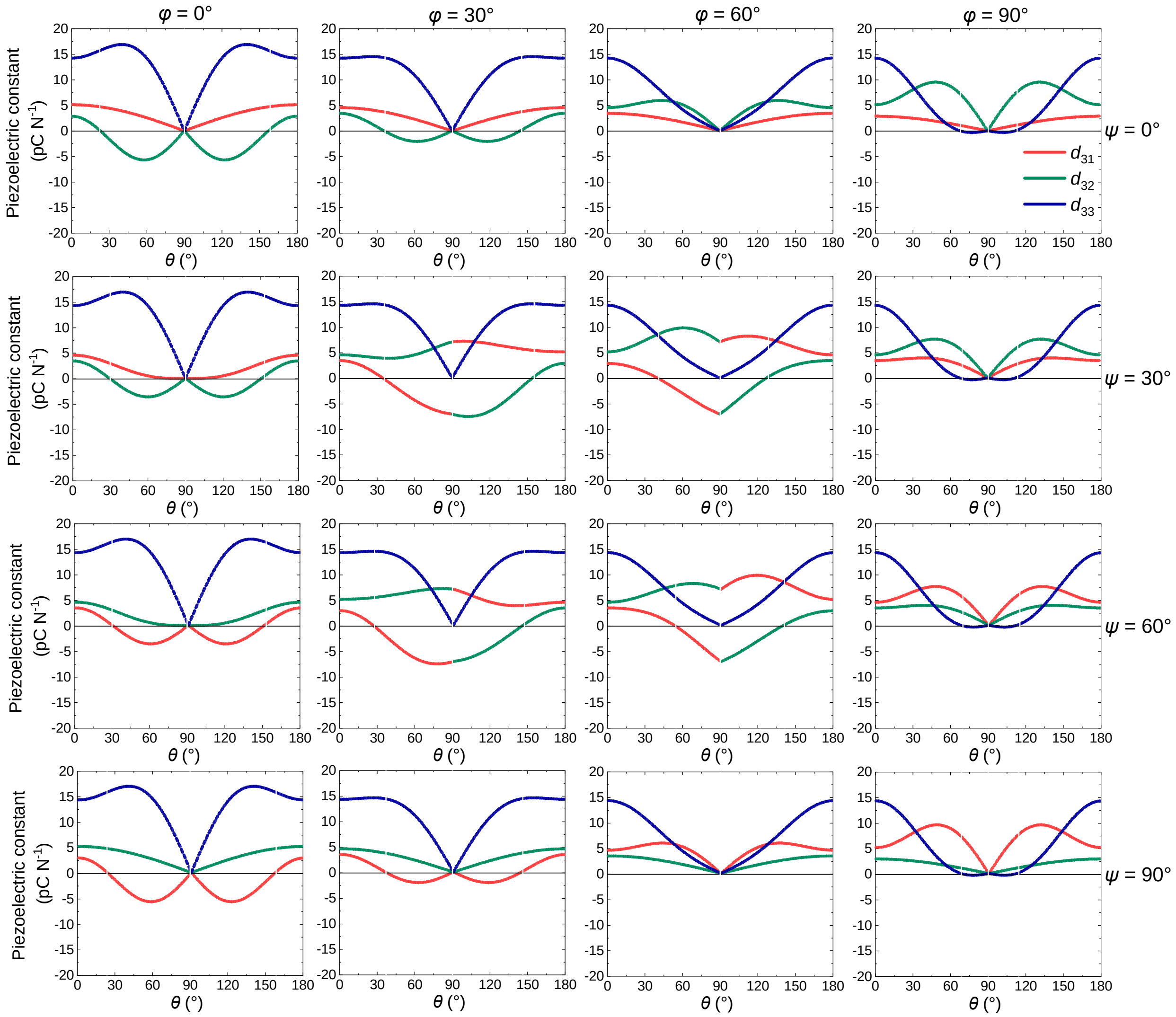

Fig. S4. Angle (θ)-dependent piezoelectric coefficients d_{31}, d_{32} and d_{33} in $CaBi_2TaO_9$ crystal. The results show that d_{31}, d_{32}, and d_{33} can keep positive value simultaneously on most low-θ region. It should be noted that the polarization can be aligned with the external electric field due to the nature of ferroelectricity, which suggests that the polycrystal ceramics should be possible to achieve the auxetic piezoelectricity.

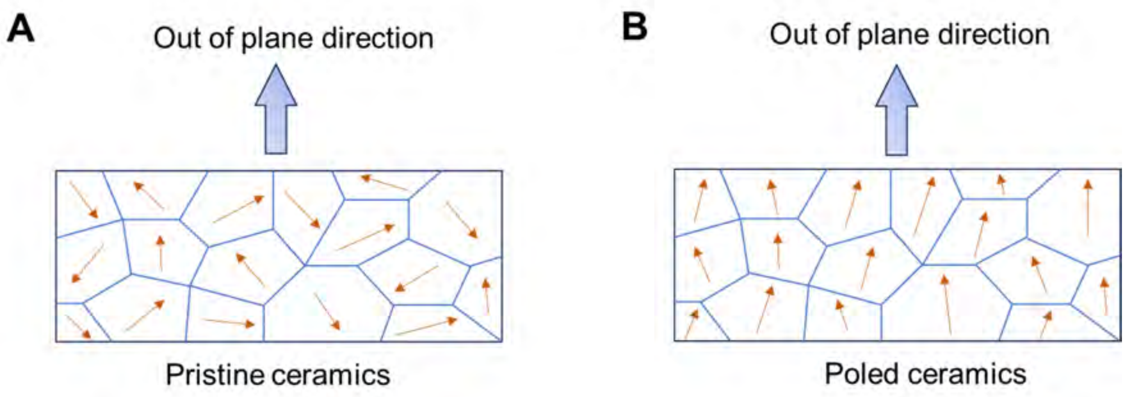

Fig. S5. Schematic illustration of domain orientation in a piezoelectric ceramic disk before and after poling. The electric field is applied along the out-of-plane direction of the ceramic disk. The angle between the out-of-plane direction and the polarization vector corresponds to the actual θ for each domain. During the poling process, the polarization vectors tend to align with the external electric field, resulting in progressively lower-θ states for individual domains after sufficient poling. Since the piezoelectric response of a polycrystalline ceramic is defined as the collective contribution of all domains, the macroscopic auxetic piezoelectric effect can therefore be preserved.

Section S4: Calculated piezoelectric constants of bismuth-layered structure ceramics.

Table S5. Predicted piezoelectric strain constants of orthorhombic bismuth-layered structure ceramics, in the unit of pC N^{-1}.

Materials	d_{31}	d_{33}	d_{15}
Bi_2WO_6	3.10	12.75	17.10
$CaBi_2Nb_2O_9$	1.94	13.85	21.01
$SrBi_2Nb_2O_9$	0.49	17.18	28.71
$BaBi_2Nb_2O_9$	-0.79	26.44	45.43
$CaBi_2Ta_2O_9$	3.44	8.82	8.72
$SrBi_2Ta_2O_9$	1.22	7.94	10.04
$BaBi_2Ta_2O_9$	2.94	12.18	13.23
$PbBi_2Ta_2O_9$	2.55	15.15	19.62
$Bi_4Ti_3O_{12}$	4.57	15.49	18.46
$CaBi_4Ti_4O_{15}$	3.47	14.96	19.40
$SrBi_4Ti_4O_{15}$	2.15	17.60	26.90
$BaBi_4Ti_4O_{15}$	0.75	23.62	43.58
$PbBi_4Ti_4O_{15}$	1.38	22.03	37.14

Section S5: Predicted piezoelectric properties at finite temperature.

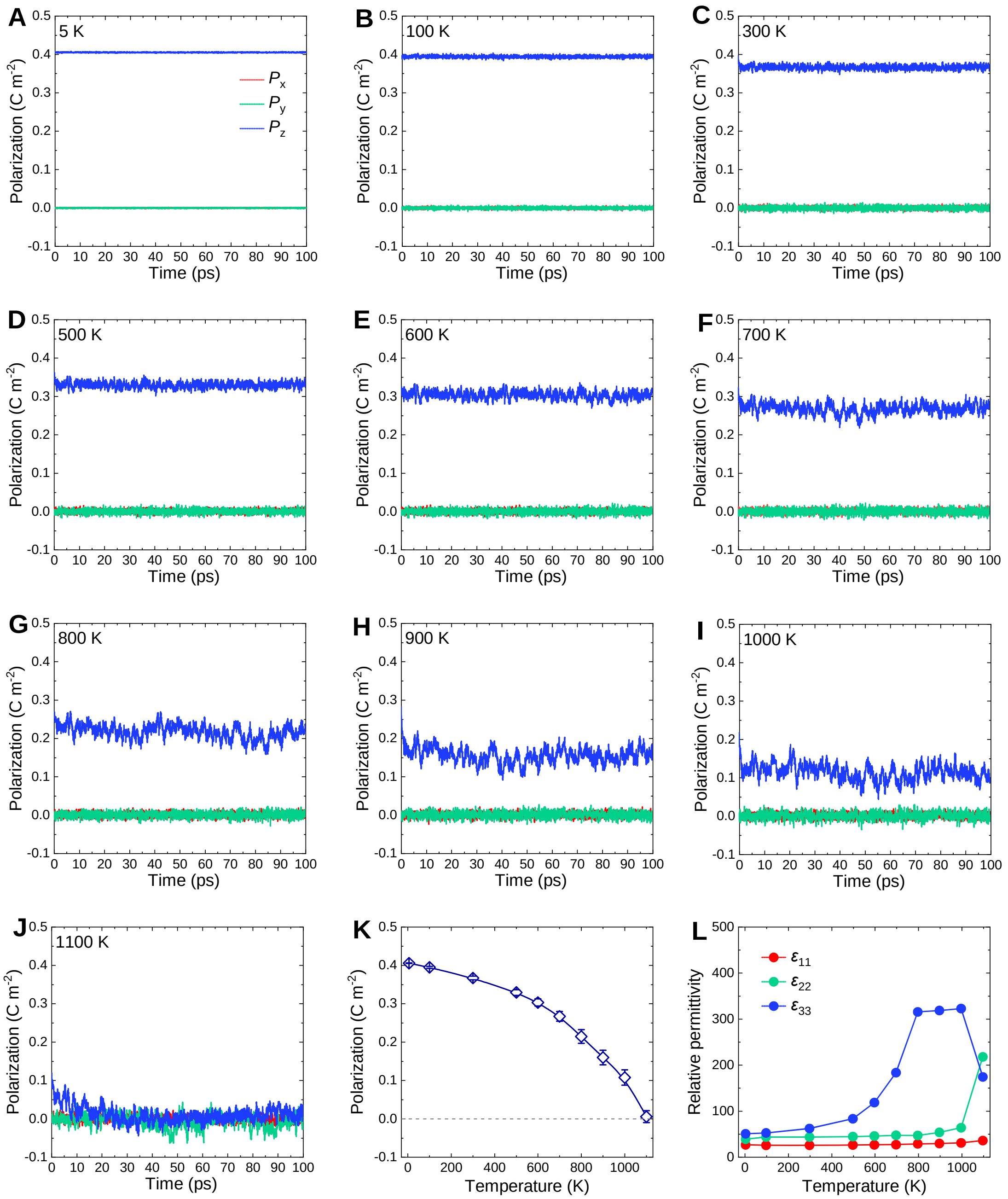

Fig. S6. Polarization and dielectric constants of $CaBi_2Ta_2O_9$ at finite temperature. (**A**)-(**J**) Time-dependent polarization at difference temperature for $CaBi_2Ta_2O_9$ based on MLFF molecular dynamics; (**K**) Temperature-dependent polarization; (**L**) Temperature-dependent relative permittivity.

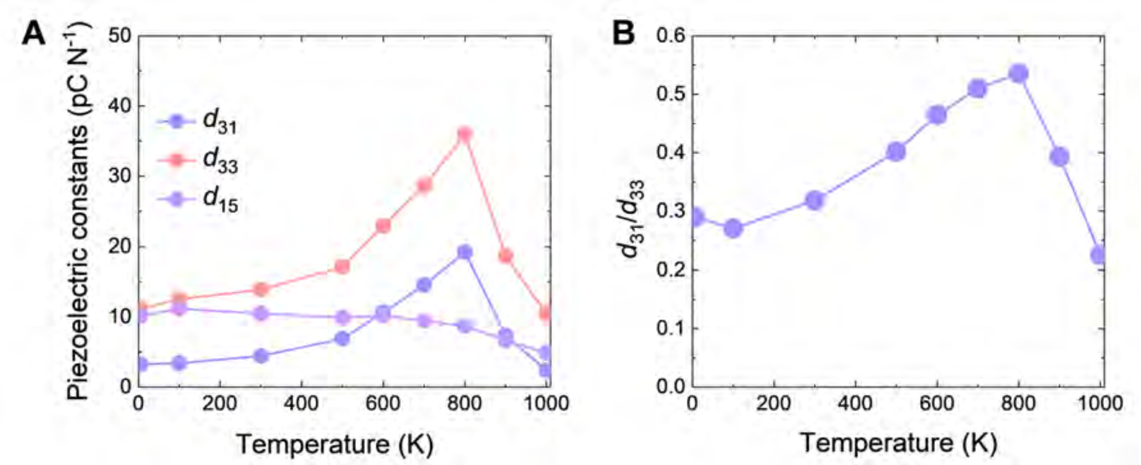

Fig. S7. Calculated temperature-dependent piezoelectric constant for $CaBi_2Ta_2O_9$ ceramics. Despite the d_{31} and d_{33} is temperature-dependent, the ratio of d_{31} and d_{33} is always positive during all simulation temperatures.

Table S6. Calculated piezoelectric strain constants of $CaBi_2Ta_2O_9$ crystal at finite temperature, in the unit of pC N^{-1}.

Temperature (K)	d_{31}	d_{32}	d_{33}	d_{24}	d_{15}
5	6.72	3.15	15.10	24.44	4.99
100	7.22	2.63	17.44	26.25	5.59
300	10.61	1.43	20.04	24.49	4.76
500	18.86	0.09	24.79	22.67	4.79
600	29.57	-0.36	33.26	22.08	5.89
700	41.31	-2.70	42.96	19.80	4.24
800	52.86	-4.25	56.54	15.69	3.14
900	27.85	-10.40	29.69	13.68	1.85
1000	20.87	-15.43	16.02	13.08	-0.56

Table S7. Calculated piezoelectric strain constants of $CaBi_2Ta_2O_9$ ceramics at finite temperature, in the unit of pC N^{-1}.

Temperature (K)	d_{31}	d_{33}	d_{15}	d_{31}/d_{15}
5	3.24	11.18	10.15	0.29
100	3.39	12.52	11.16	0.27
300	4.41	13.86	10.49	0.32
500	6.89	17.15	9.90	0.40
600	10.64	22.89	10.20	0.47
700	14.56	28.55	9.41	0.51
800	19.23	35.90	8.70	0.54
900	7.34	18.66	6.66	0.39
1000	2.36	10.44	4.86	0.23

Section S6: Piezoelectric characterization of $CaBi_2Ta_2O_9$ and PZT ceramics

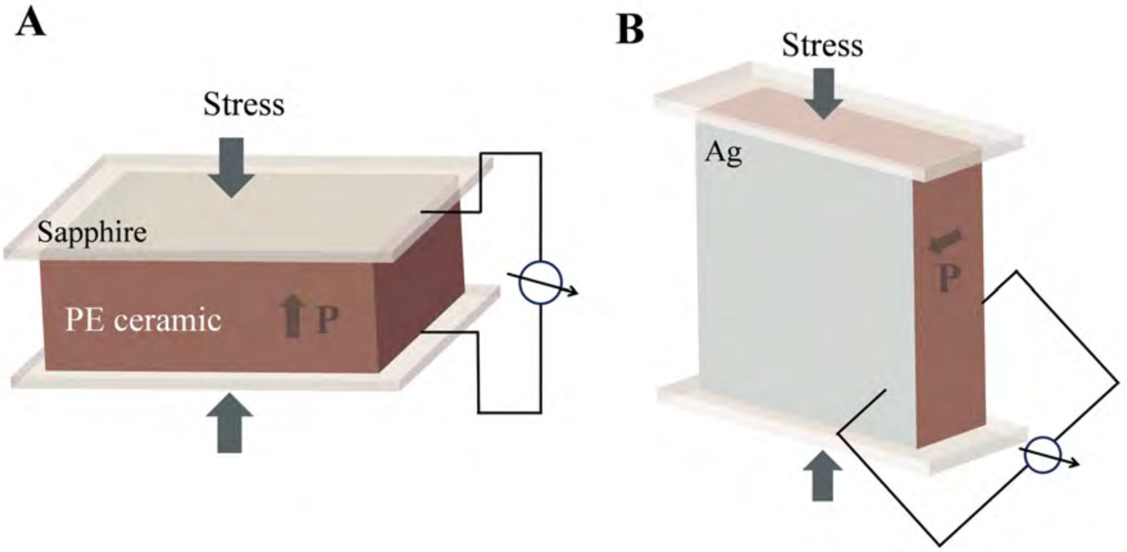

Fig. S8. Schematics showing the measurement geometry to characterize direct piezoelectric coefficients. (**A**) the d_{33} measurement setup and (**B**) the d_{31} measurement setup.

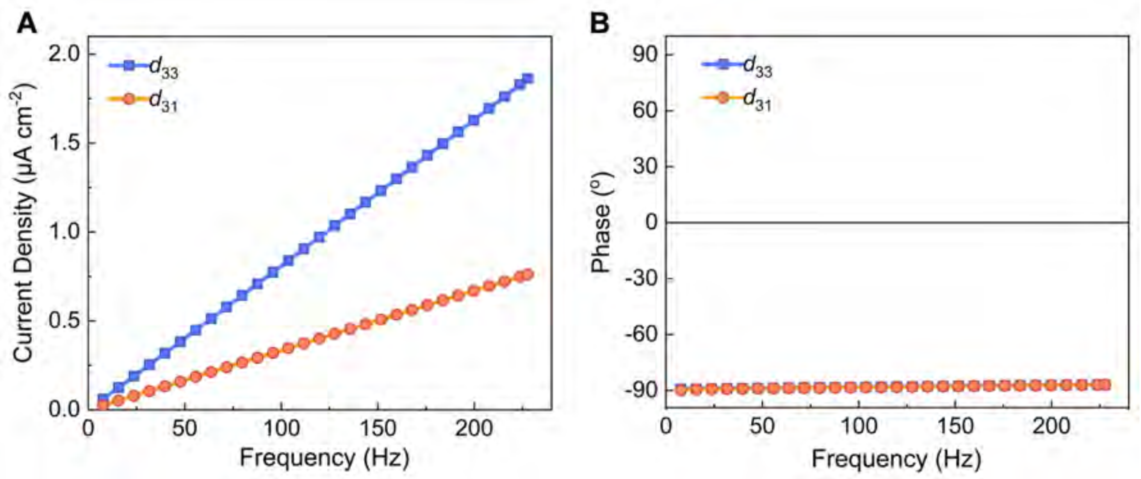

Fig. S9. Frequency dependent direct piezoelectric response of modified $CaBi_2Ta_2O_9$ ceramic. (**A**) the amplitude and (**B**) phase of the current density. The stress for measurement is normalized to 1 MPa. Based on the relationship $J_3 = 2\pi f d_{3i}\sigma_i$, where f is the frequency of applied stress, J_3 is the amplitude of current density, σ_i is the amplitude of stress, the piezoelectric coefficients d_{33} and d_{31} can be calculated as 12.8 pC N^{-1} and 5.4 pC N^{-1}, respectively.

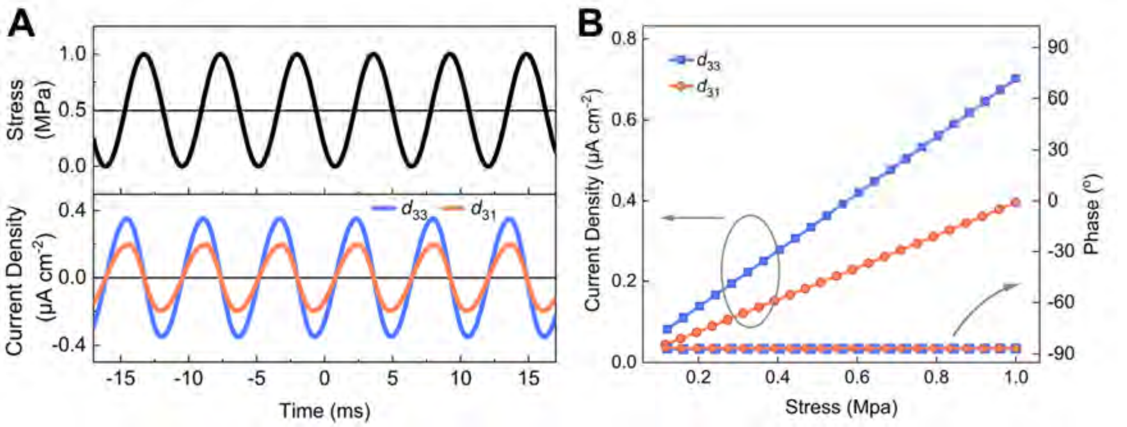

Fig. S10. Direct piezoelectric characterization of pure $CaBi_2Ta_2O_9$ ceramics with piezoelectric auxeticity. (**A**) Waveform of applied sinusoidal compressive stress (top panel), and the induced current density (bottom panel) for Ag/$CaBi_2Ta_2O_9$/Ag capacitors, with stress respectively applied parallel (d_{33}) and perpendicular (d_{31}) to the direction of polarization. (**B**) Stress-dependent short-circuit current density induced by piezoelectric d_{33} and d_{31} with the measurement frequency of 177.7 Hz.

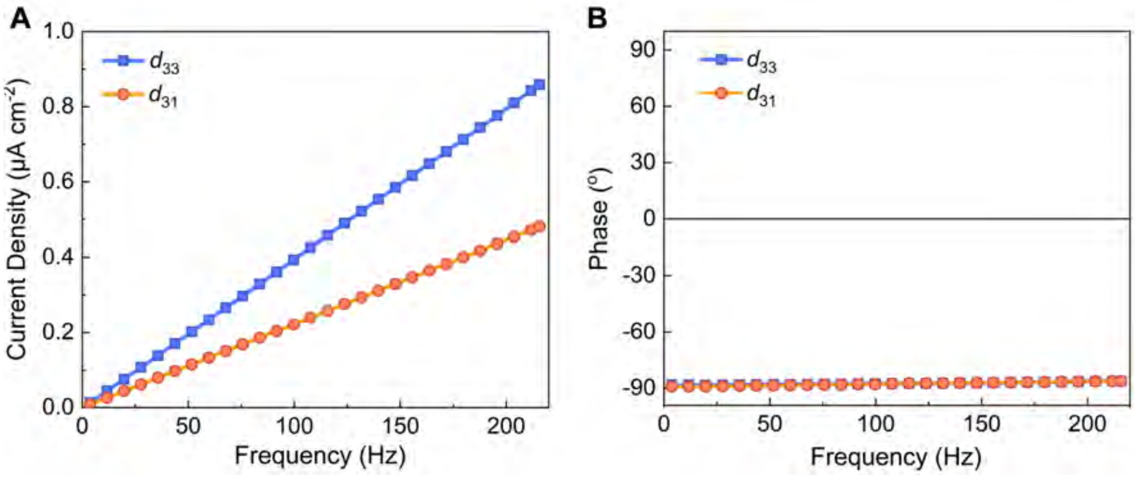

Fig. S11. Frequency dependent direct piezoelectric response of pure $CaBi_2Ta_2O_9$ ceramic. (**A**) the amplitude and (**B**) phase of the current density. The stress for measurement is normalized to 1 MPa. Based on the relationship $J_3 = 2\pi f d_{3i}\sigma_i$, where f is the frequency of applied stress, J_3 is the amplitude of current density, σ_i is the amplitude of stress, the piezoelectric coefficients d_{33} and d_{31} can be calculated as 6.2 pC N^{-1} and 3.5 pC N^{-1}, respectively.

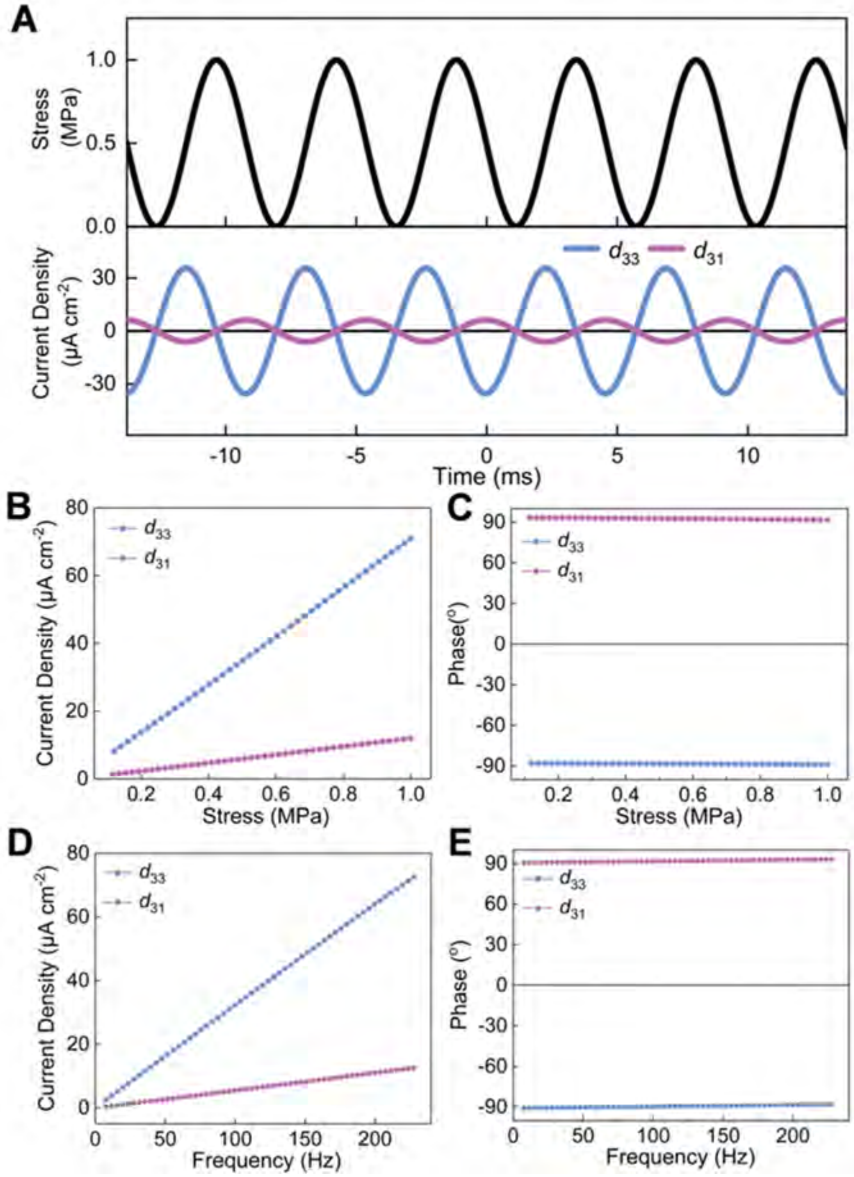

Fig. S12. Direct piezoelectric characterization of PZT-5h ceramic with conventional piezoelectric effect. (**A**) Waveform of applied sinusoidal compressive stress (top panel), and induced current density waveform (bottom panel) for Ag/PZT-5h/Ag capacitors, with stress applied parallel (d_{33}, cyan curve) and perpendicular (d_{31}, purple curve) to the direction of polarization. (**B**) Stress-dependent amplitude and (**C**) Stress-dependent phase of the current density output with a frequency of 217.7 Hz. (**D**) Frequency-dependent amplitude and (**e**) frequency-dependent phase of the current density output. The stress here is normalized to 1 MPa. The piezoelectric coefficients d_{33} and d_{31} can be calculated as 490 pC N^{-1} and -89 pC N^{-1}, respectively.

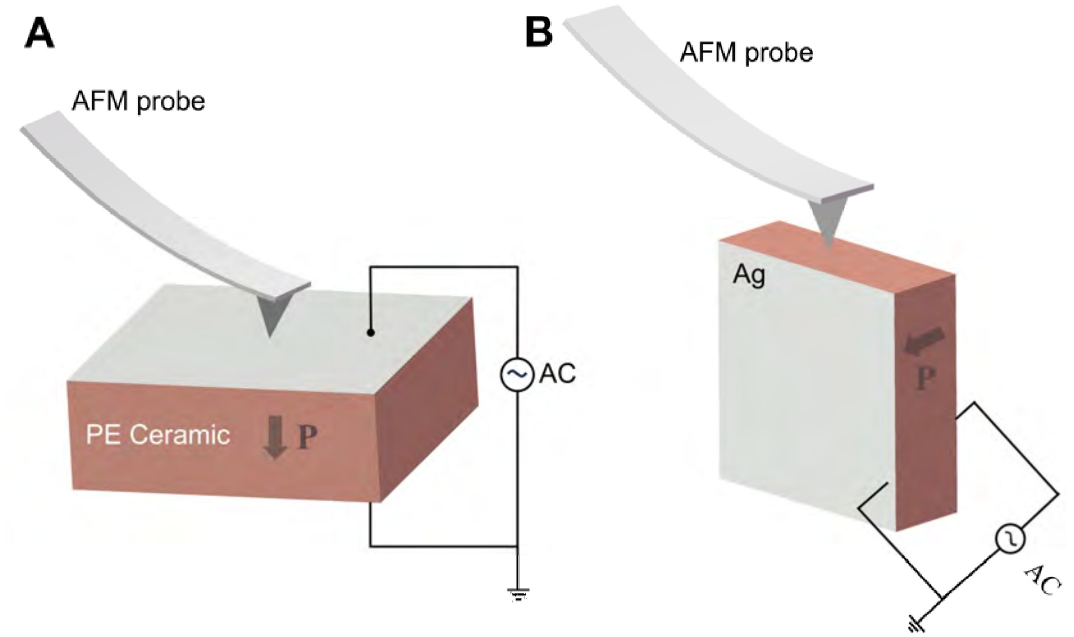

Fig. S13. Schematics showing the measurement geometry to characterize converse piezoelectric coefficients. (**A**) The d_{33} measurement setup and (**B**) the d_{31} measurement setup.

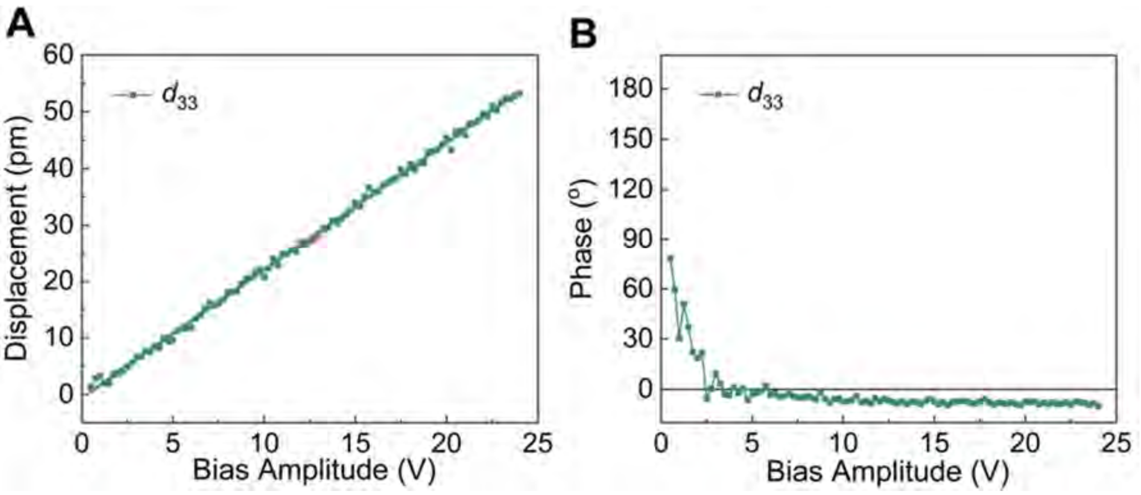

Fig. S14. Converse piezoelectric characterization of X-cut quartz single crystal using AFM system. (**A**)Voltage-dependent displacement amplitude and (**B**) voltage-dependent phase measured at a frequency of 17.777khz. The extracted piezoelectric coefficient d_{33} can be calculated as 2.26 pm V^{-1}, which is in good agreement with the intrinsic value of quartz.

This result demonstrates that our AFM setup is capable of accurately quantifying the piezoelectric coefficient of materials.

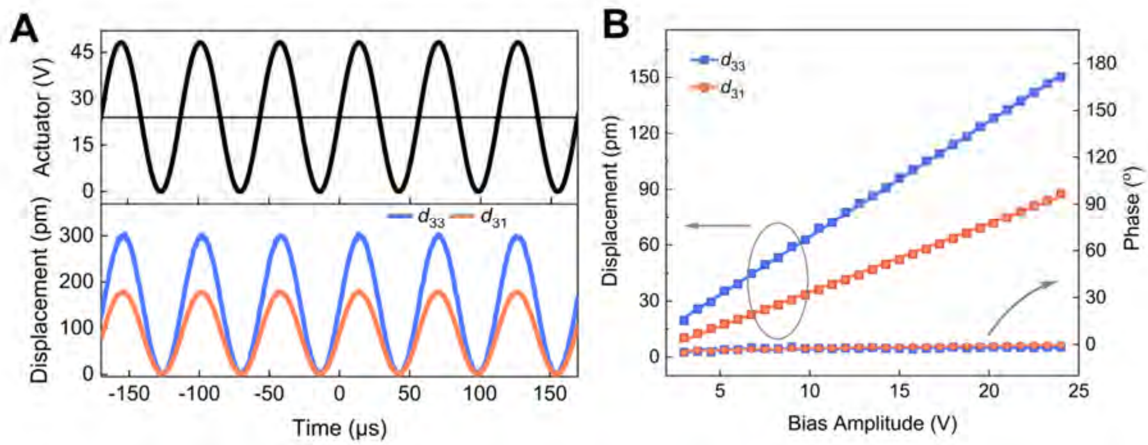

Fig. S15. Converse piezoelectric characterization of pure $CaBi_2Ta_2O_9$ ceramics with piezoelectric auxeticity. (**A**) Waveform of the applied sinusoidal bias voltage (top panel), and the induced displacement waveform (bottom panel) for Ag/$CaBi_2Ta_2O_9$/Ag capacitors, with the induced displacement parallel (d_{33}) and perpendicular (d_{31}) to the direction of polarization. (**B**) Bias amplitude dependence of amplitude and phase of the longitudinal (i.e. d_{33}) and transverse (d_{31}) displacement with the measurement frequency of 17.777 kHz.

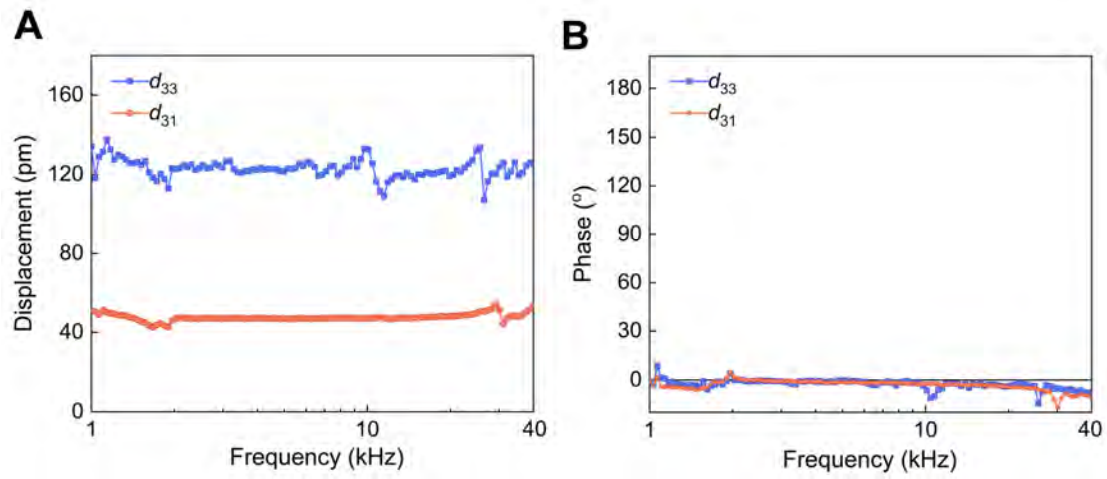

Fig. S16. Frequency-dependent converse piezoelectric response of modified $CaBi_2Ta_2O_9$ ceramics. (**A**) The amplitude and (**B**) the phase of the displacement as a function of the frequency. The bias amplitude is set as 9 V.

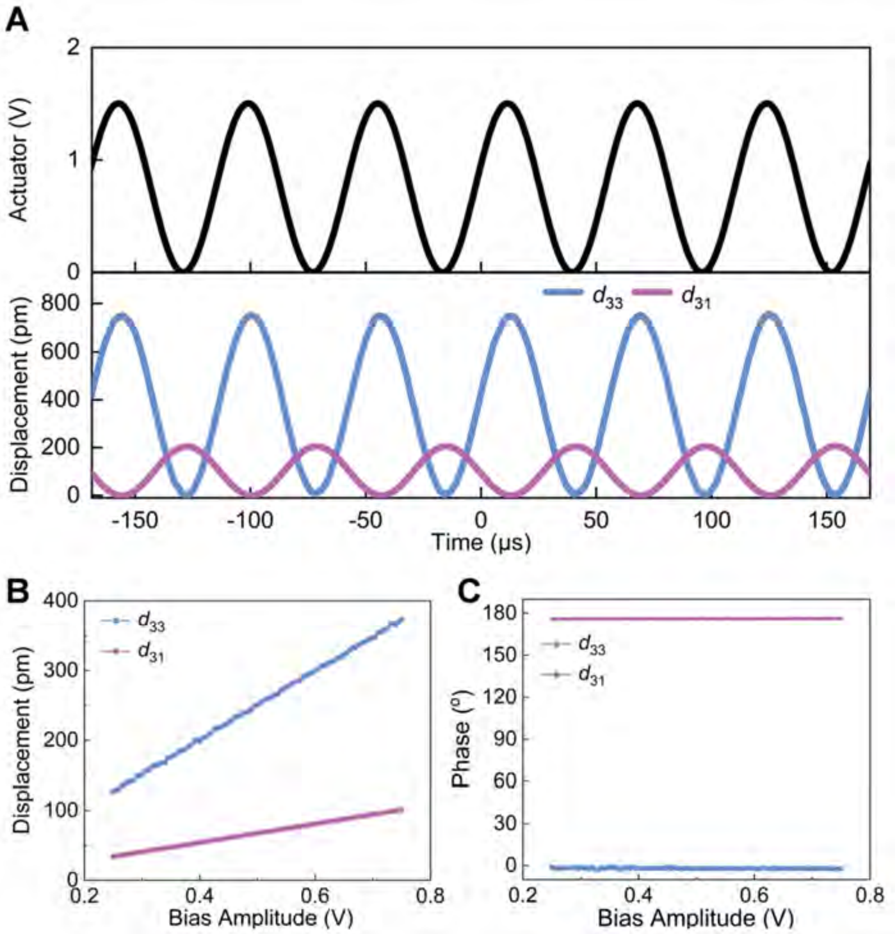

Fig. S17. Converse piezoelectric characterization of PZT-5h ceramics with conventional piezoelectric effect. (**A**) Waveform of applied a.c. voltage (top panel), and induced displacement waveform (bottom panel) for Ag/PZT-5h/Ag capacitors, showing displacement parallel (d_{33}, cyan curve) and perpendicular (d_{31}, purple curve) to the direction of polarization. (**B**) Voltage-dependent amplitude and (**C**) Voltage-dependent phase of the displacement output, with a frequency of 17.777 kHz.

Section S7: Mechanism of abnormal d_{31} in $CaBi_2Ta_2O_9$.

Table S8. Calculated structural parameters of orthorhombic $CaBi_2Ta_2O_9$.

Space group	Lattice parameters (Å)	Atomic coordinate			
		Atom	*x*	*y*	*z*
*Cmc*2$_1$	*a* = 24.985	Ca (4*a*)	0.0000	0.7559	0.0002
	b = 5.424	Bi (8*b*)	0.8011	0.2239	0.5415
	c = 5.473	Ta (8*b*)	0.5817	0.2473	0.4636
		O1 (8*b*)	0.7495	0.0101	0.2687
		O2 (8*b*)	0.6551	0.3281	0.4539
		O3 (8*b*)	0.4135	0.0558	0.1476
		O4 (8*b*)	0.9386	0.0379	0.2352
		O5 (4*a*)	0.0000	0.6702	0.4291

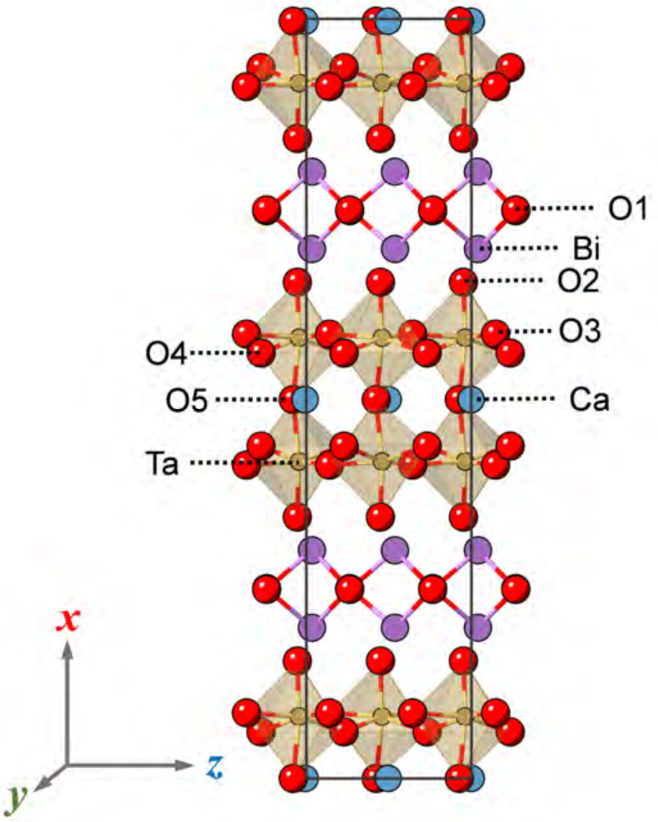

Fig. S18. Crystal structure diagram with atom labels. $CaBi_2Ta_2O_9$ with *Cmc*2_1 symmetry.

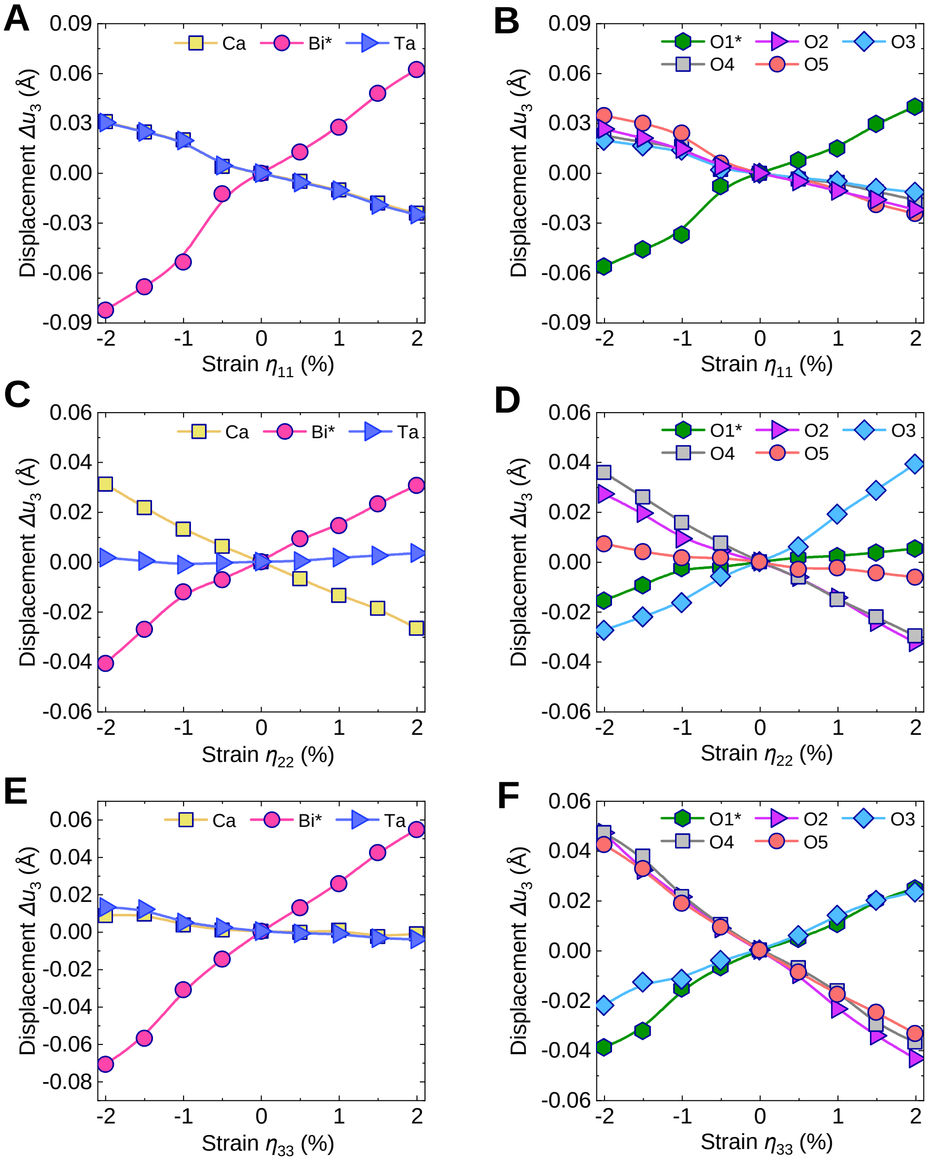

Fig. S19. Displacements Δu_i of different atoms in $CaBi_2Ta_2O_9$ under the uniaxial strain. Here, the displacement is defined as the $\Delta u_i = a_i \Delta w_i$, where w is the internal atomic coordinate and a is the cell parameter.

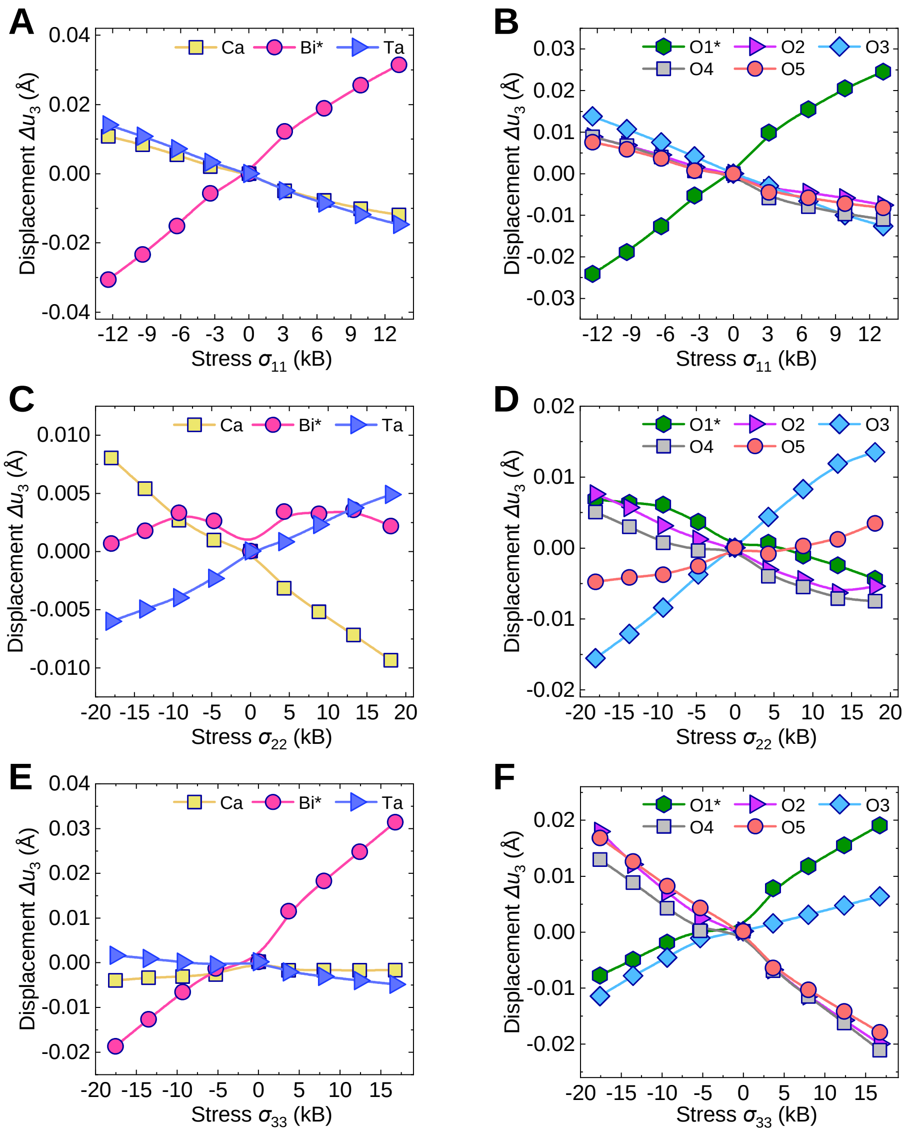

Fig. S20. Displacements Δu_i of different atoms in $CaBi_2Ta_2O_9$ under the uniaxial stress. Here, the displacement is defined as the $\Delta u_i = a_i \Delta w_i$, where w is the internal atomic coordinate and a is the cell parameter.

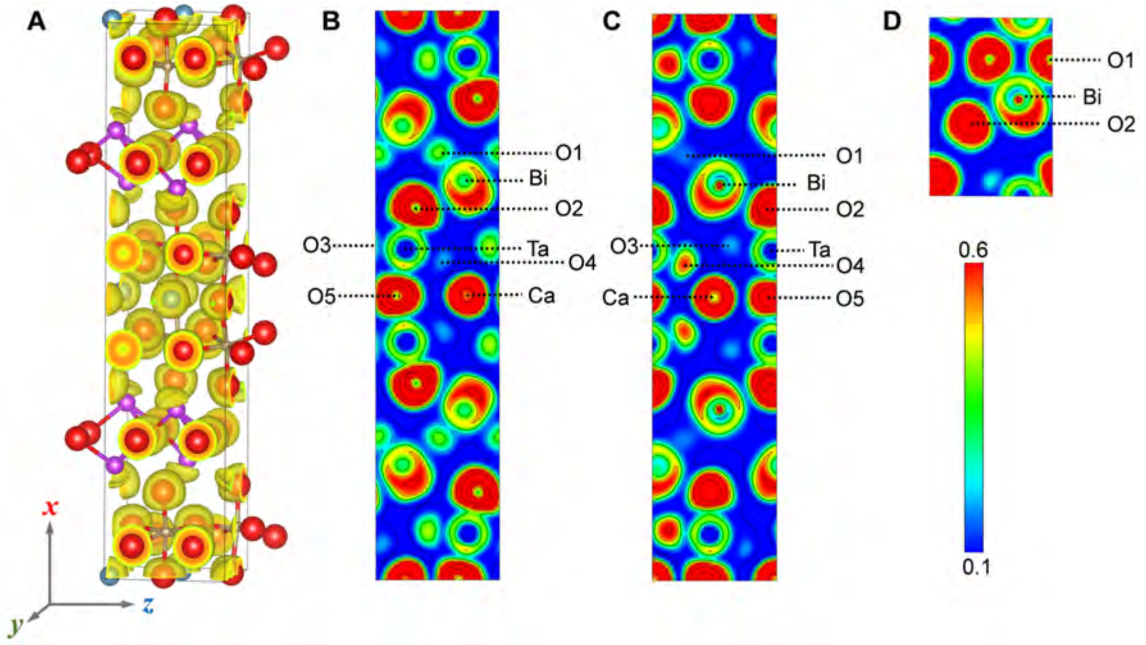

Fig. S21. Electron localization function (ELF) of $CaBi_2Ta_2O_9$. (**A**) 3d ELF of $CaBi_2Ta_2O_9$, where the isosurface level is set to 0.6; (**B**) 2d ELF of (001) plane at z = 5.22 Å; (**C**) 2d ELF of (010) plane at y = 2.92 Å; (**D**) 2d section involved Bi-O bond. It should be noted that the bond between Ca and O3 is completely ionic, and the bonds between Ta and O2 or O3 and between Bi and O1 is most ionic but involved small partly covalent. The bond between Bi and O2 is special, the ELF near Bi atoms in Fig. S18D is at the lower right position of Bi atom rather than at the middle of Bi-O2 bond. The result indicates that the bond between Bi and O2 is still ionic but charge transfer is weak from Bi to O2, suggesting the bond between Bi and O2 should be not strong.

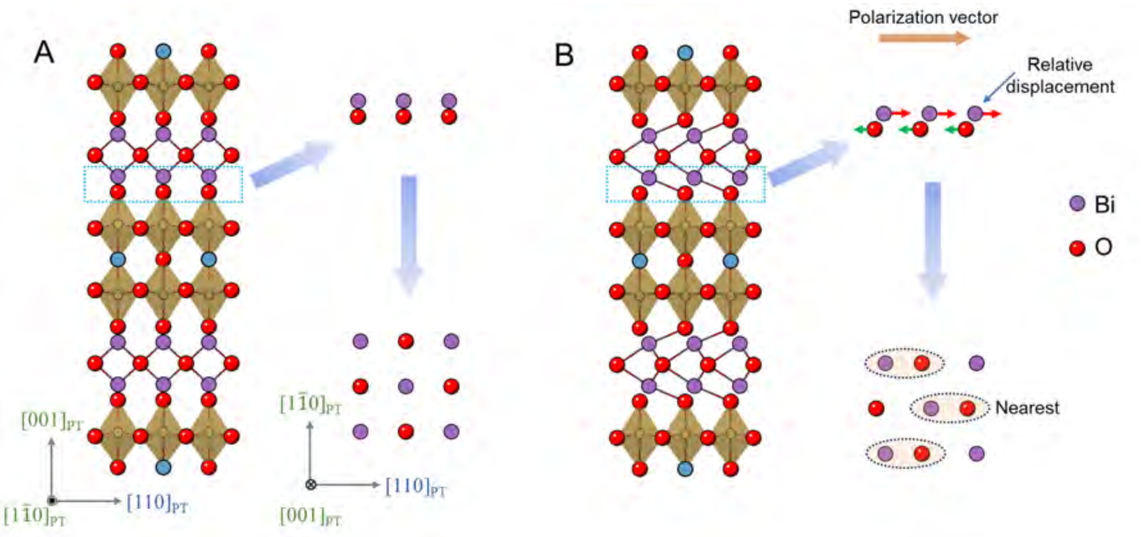

Fig. S22. Schematic diagrams of interface between Bi_2O_2 layer and perovskite block. (**A**) paraelectric $CaBi_2Ta_2O_9$, and **(B**) ferroelectric $CaBi_2Ta_2O_9$. Purple spheres represent Bi atoms and red spheres represent O atoms. Their lower-right panels show the top view of the interface between the Bi_2O_2 layer and the perovskite block along the $[001]_{PT}$ direction. The subscript PT denotes the pseudo-tetragonal lattice form. The directions $[001]_{PT}$, $[1\bar{1}0]_{PT}$, and $[110]_{PT}$ corresponding to [100], [010], and [001] in the *Cmc*2_1 phase, respectively. The structure shows that the Bi atoms in the Bi_2O_2 layer are staggered relative to the O atoms in the outer layer of the perovskite block. It should be noted that the Bi atom is closely adjacent to the outer oxygen atom of the perovskite block (denoted as the O2 atom) along the $[110]_{PT}$ direction. The Bi–O2 bond therefore plays a crucial role in determining the interlayer spacing.

Section S8: Mechanism of abnormal d_{31} in Bi_2WO_6.

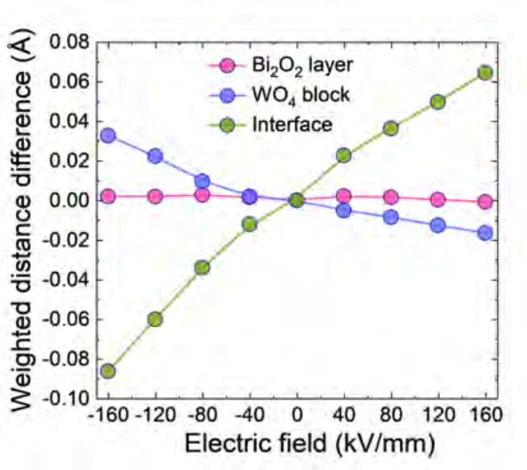

Fig. S23. Weighted distance evolution of Bi_2WO_6 under external electric field. Weighted distances of different part along *x* direction under the external electric field E_3. One *Bba*2 unit cell of Bi_2WO_6 contain 2 Bi_2O_2 layers, 2 WO_4 blocks, and 4 interfaces between Bi_2O_2 and WO_4, so the weighted factors are 2, 2, and 4, respectively. The results show that the Bi_2O_2 layer is very hard to be compressed or expanded, and thickness of Bi_2O_2 layer has barely changed. The perovskite block, WO_4, follows the law of conventional perovskite materials, such as $KNbO_3$ in the *x* direction, that is, the thickness of WO_4 block is decreased with the increased electric field. Hence, it can be found that the lateral expansion of Bi_2WO_6 can be attributed to the increased distance between Bi_2O_2 and WO_4 in *x* direction due to the electricity-induced lattice mismatch.

Section S9: Piezoelectric characterization of other bismuth-layered structure ceramics.

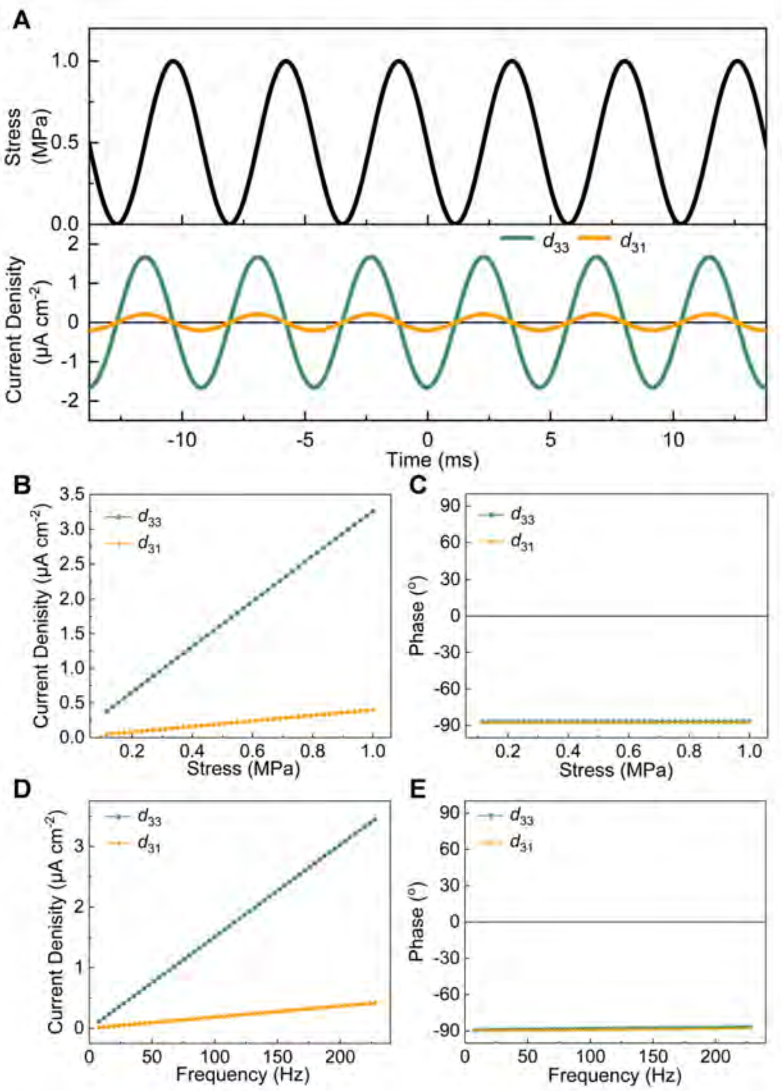

Fig. S24. Direct piezoelectric characterization of modified $CaBi_4Ti_4O_{15}$ ceramics with auxetic piezoelectric effect. (**A**) Waveform of applied sinusoidal compressive stress (top panel), and induced current density waveform (bottom panel) for Ag/$CaBi_4Ti_4O_{15}$/Ag capacitors, with stress applied parallel (d_{33}, green curve) and perpendicular (d_{31}, orange curve) to the direction of polarization. (**B**) Stress-dependent amplitude and (**C**) stress-dependent phase of the current density output, with a frequency of 217.7 Hz. (**D**) Frequency-dependent amplitude and (**E**) frequency-dependent phase of the current density output. The stress here is normalized to 1 MPa. The piezoelectric coefficients d_{33} and d_{31} can be calculated as 23.7 pC N^{-1} and 2.8 pC N^{-1}, respectively.

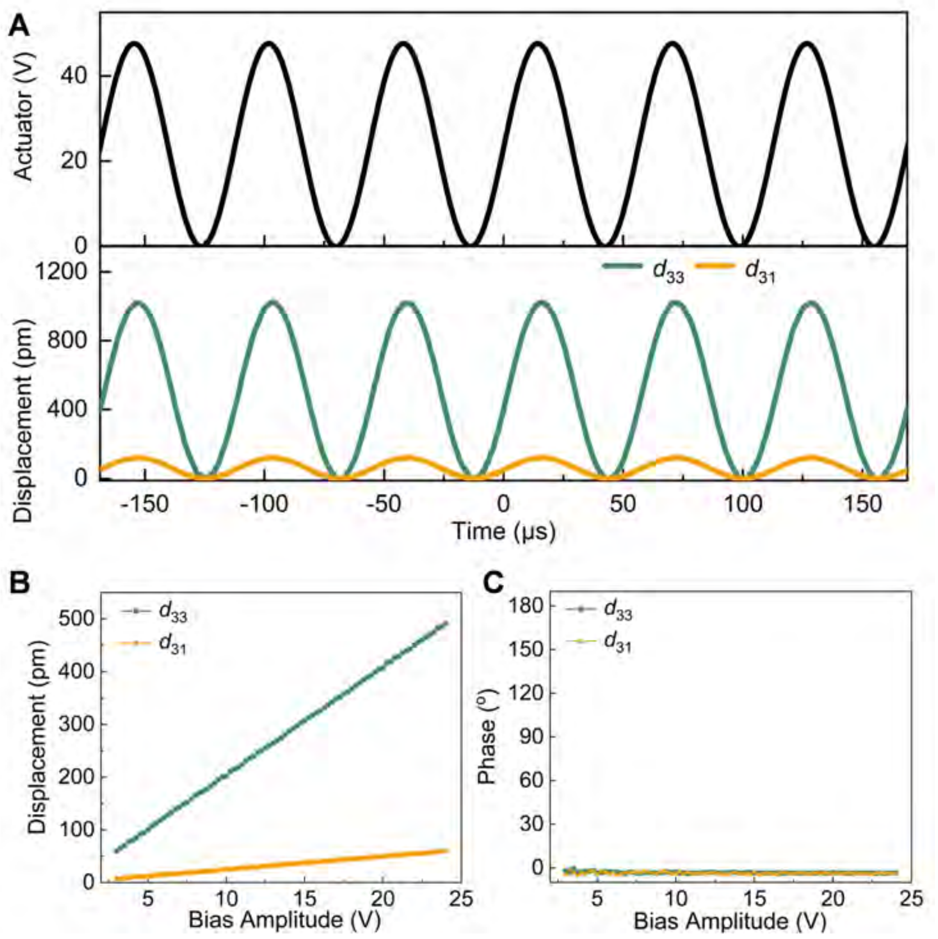

Fig. S25. Converse piezoelectric characterization of modified $CaBi_4Ti_4O_{15}$ ceramics with auxetic piezoelectric effect. (**A**) Waveform of applied a.c. voltage (top panel), and induced displacement waveform (bottom panel) for Ag/$CaBi_4Ti_4O_{15}$/Ag capacitors, showing displacement parallel (d_{33}, green curve) and perpendicular (d_{31}, orange curve) to the direction of polarization. (**B**) Voltage-dependent amplitude and (**C**) Voltage-dependent phase of the displacement output, with a frequency of 17.777 kHz.

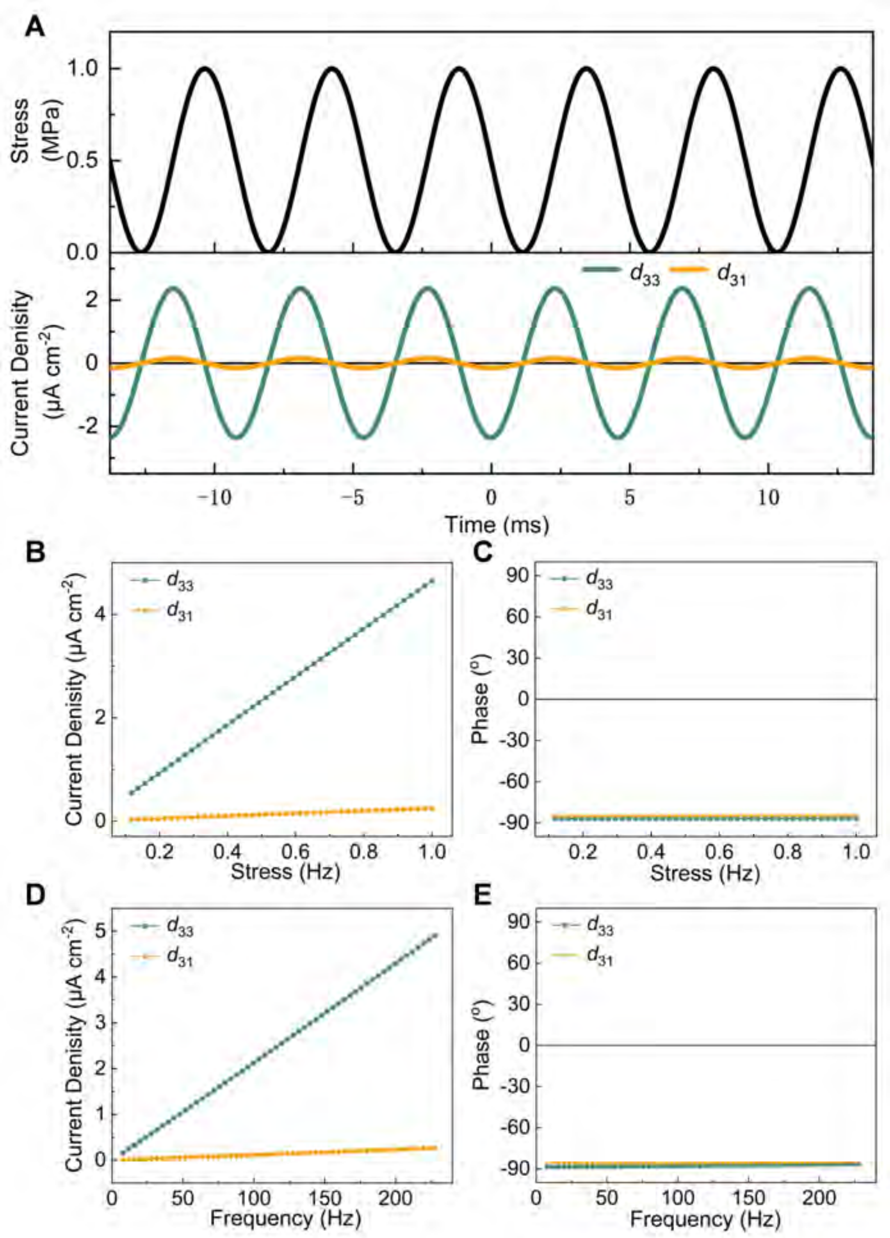

Fig. S26. Direct piezoelectric characterization of modified $Bi_4Ti_3O_{12}$ ceramics with auxetic piezoelectric effect. (**A**) Waveform of applied sinusoidal compressive stress (top panel), and induced current density waveform (bottom panel) for Ag/$Bi_4Ti_3O_{12}$/Ag capacitors, with stress applied parallel (d_{33}, green curve) and perpendicular (d_{31}, orange curve) to the direction of polarization. (**B**) Stress-dependent amplitude and (**C**) stress-dependent phase of the current density output, with a frequency of 217.7 Hz. (**D**) Frequency-dependent amplitude and (**E**) Frequency-dependent phase of the current density output. The stress here is normalized to 1 MPa. The piezoelectric coefficients d_{33} and d_{31} can be calculated as 33.8 pC N^{-1} and 1.6 pC N^{-1}, respectively.

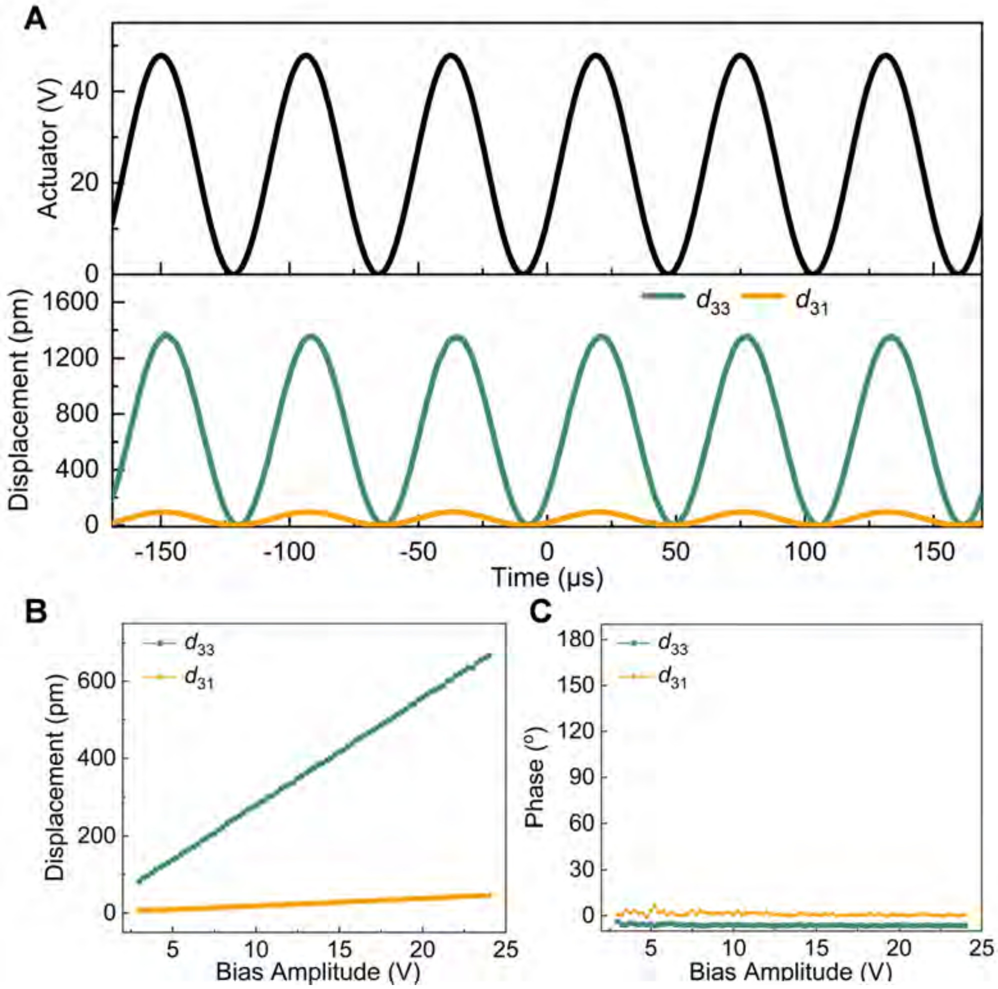

Fig. S27. Converse piezoelectric characterization of modified $Bi_4Ti_3O_{12}$ ceramics with auxetic piezoelectric effect. (**A**) Waveform of applied a.c. voltage (top panel), and induced displacement waveform (bottom panel) for Ag/$Bi_4Ti_3O_{12}$/Ag capacitors, showing displacement parallel (d_{33}, green curve) and perpendicular (d_{31}, orange curve) to the direction of polarization. (**B**) Voltage-dependent amplitude and (**C**) voltage-dependent phase of the displacement output, with a frequency of 17.777 kHz.

Table S9. Experimentally measured Curie temperatures and piezoelectric coefficients together with their uncertainties for the investigated materials.

Materials	Curie Temp. T_c (°C)	Direct d_{33} (pC N^{-1})	Direct d_{31} (pC N^{-1})	Converse d_{33} (pm V^{-1})	Converse d_{31} (pm V^{-1})
$CaBi_2Ta_2O_9$	906±2	12.8±0.8	5.4±0.3	12.6±0.8	5.3±0.7
Pure $CaBi_2Ta_2O_9$	917±2	6.2±0.6	3.5±0.2	6.2±0.5	3.4±0.3
$Bi_4Ti_3O_{12}$	796±2	33.8±1.0	1.6±0.2	28.4±0.8	1.6±0.2
$CaBi_4Ti_4O_{15}$	532±1	23.7±1.0	2.8±0.2	20.0±0.5	2.7±0.3

Possible systematic errors in both the direct and converse piezoelectric measurements were carefully considered and minimized through experimental control and calibration.

For the direct piezoelectric measurements, the measured values may be affected by the preload force and by non-uniform compressive stress if the top and bottom surfaces of the sample are not strictly parallel. To minimize these effects, the preload force in our setup is precisely monitored using a calibrated force sensor to ensure consistent loading conditions. In addition, an improved polishing tool was used to ensure that the top and bottom surfaces of the samples are strictly parallel, promoting uniform stress during testing.

For the converse piezoelectric measurements based on AFM, potential sources of systematic error include uncertainties in the InOLS, electrostatic force differences between the conductive tip and the sample surface, and environmental electromagnetic or vibration noise. To reduce these effects, force–distance curves were measured prior to each test point to calibrate the InOLS, conductive tips were used to maintain equipotential conditions with the sample electrode, and vibration isolation and electromagnetic shielding were implemented.

Section S10: Characterization of clamped piezoelectric ceramics.

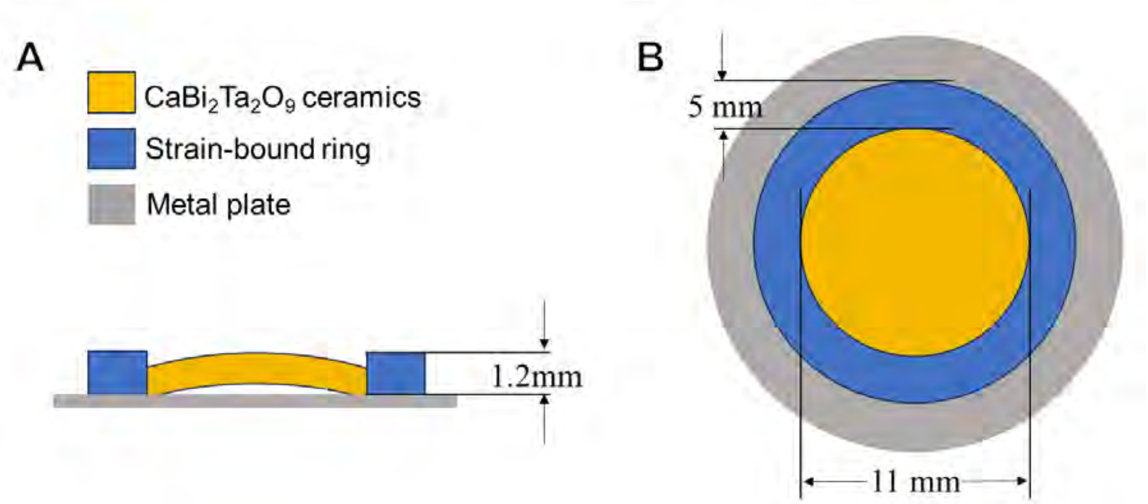

Fig. S28. Schematic diagram of designed $CaBi_2Nb_2O_9$ ceramics with fixed transverse strain. (**A**) Left view of the longitudinal section. (**B**) Top view.

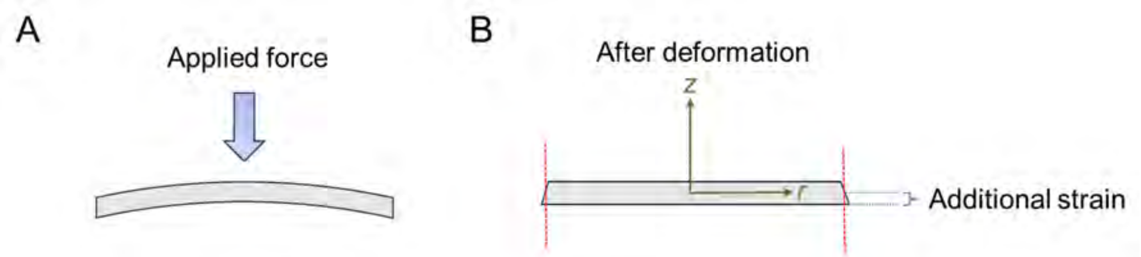

Fig. S29. Illustration of the deformation of the curved ceramic under an applied external force. The curved ceramic is loaded at the apex. Owing to the constraint imposed by the rigid ring, the transverse strain is restricted, leading to the development of additional radial stress in the cylindrical coordinate system $\{r, \theta, z\}$ during deformation. This mechanical confinement couples with the bending deformation and generates significantly larger in-plane stress than that in a flat disk. Under small-deflection conditions, the neutral plane undergoes negligible length change and can therefore be approximated as rigid, while the additional stress is mainly concentrated in the lower region of the ceramic.

For the flat ceramic disk, constraining the transverse strain already leads to an enhancement of the effective longitudinal piezoelectric coefficient due to the positive transverse piezoelectric coefficient $d_{31} > 0$. The transverse and longitudinal strains for a flat piezoelectric ceramic,

$$\eta_1 = S_{11}\sigma_1 + S_{12}\sigma_2 + d_{31}E_3 \tag{S22}$$

$$\eta_3 = S_{13}(\sigma_1 + \sigma_2) + d_{33}E_3 \tag{S23}$$

which S is the elastic compliance constant, σ is the stress ($\sigma_1 = \sigma_2$ for isotropic within the plane), E is the electric field. At the fixed strain $\eta_1 = 0$, we can resolve

$$\sigma_1 = -\frac{d_{31}E_3}{S_{11} + S_{12}} \tag{S24}$$

In this case, the effective longitudinal piezoelectric constant, $d_{33,\text{eff}}$, can be obtained

$$d_{33,eff} = \frac{\eta_3}{E_3} = d_{33} - \frac{2S_{13}}{S_{11} + S_{12}} d_{31} \tag{S25}$$

For conventional piezoelectric materials, the $S_{13} < 0$ and $d_{31} < 0$, suggesting that $d_{33,\text{eff}}$ is smaller than the original d_{33}. However, for our Aurivillius ceramics with auxetic piezoelectricity, $d_{31} > 0$ lead to a higher $d_{33,\text{eff}}$ than original d_{33}. However, the $S_{13}/(S_{11}+S_{12})$ of Aurivillius ceramics is about -0.31. Hence, despite by fixing the transverse strain of flat ceramic disk can enhance the effective d_{33}, but the overall magnitude of the improvement remains limited.

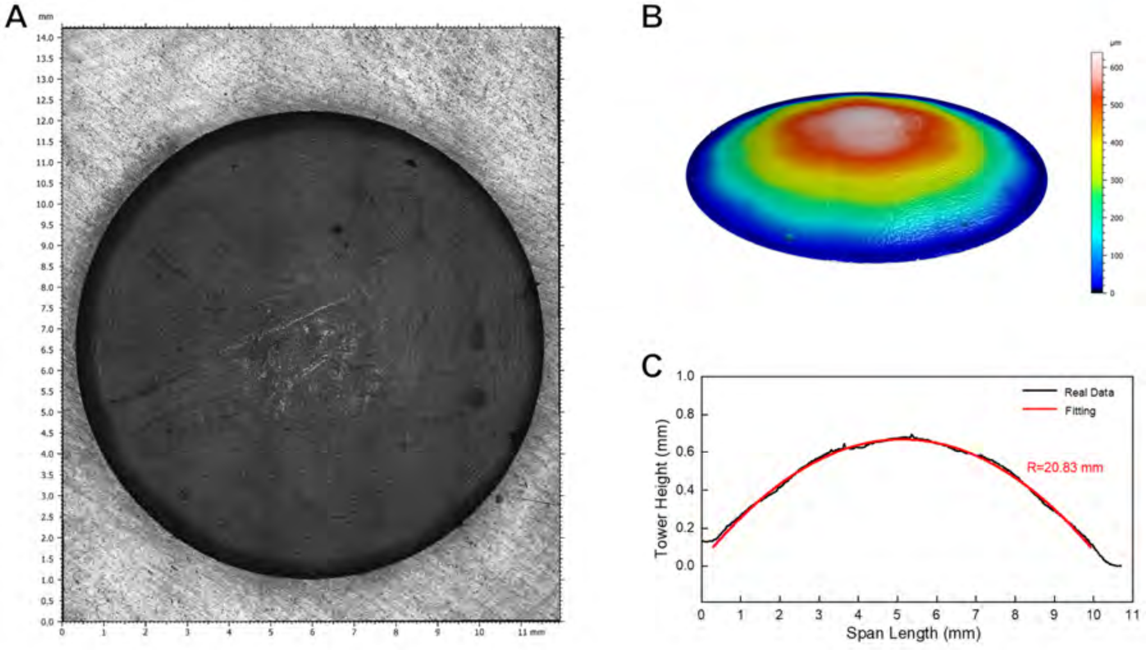

Fig. S30. Curvature characterization of designed $CaBi_2Nb_2O_9$ ceramics. (A) Optical photograph. **(B)** Surface image with colormap by height. **(C)** Fitting result of radius**.**

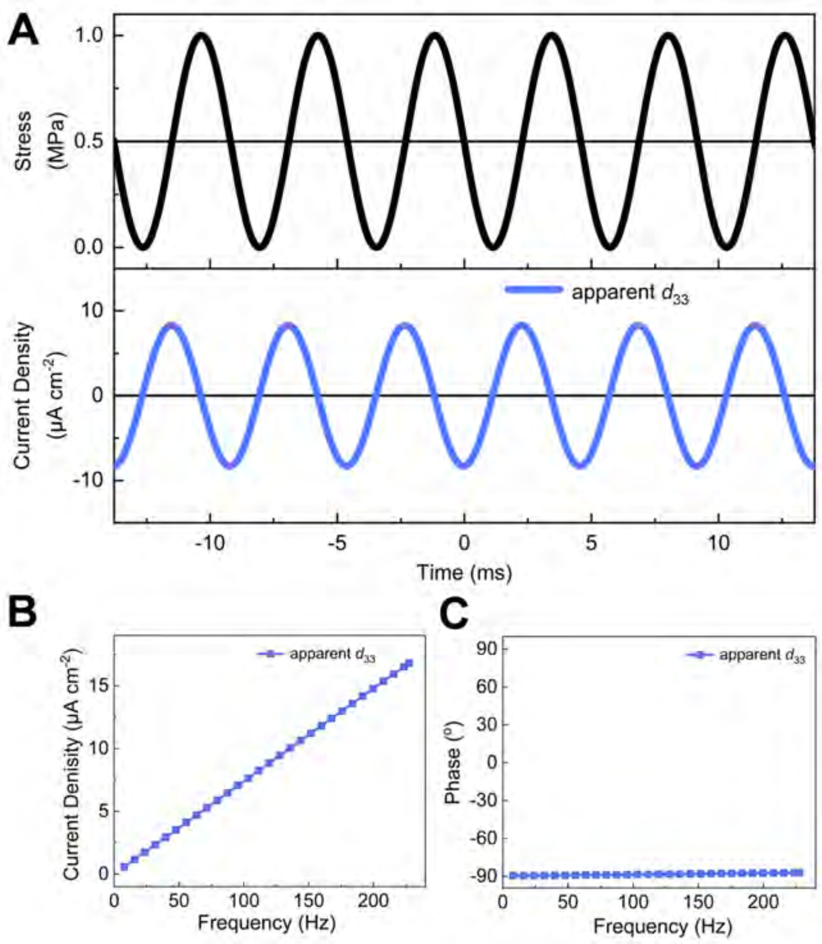

Fig. S31. Direct piezoelectric characterization of clamped $CaBi_2Nb_2O_9$ ceramic. (**A**) Waveform of applied sinusoidal compressive stress (top panel), and induced current density waveform (bottom panel) for Ag/clamped $CaBi_2Nb_2O_9$/Ag capacitors, with stress applied parallel (d_{33}, blue curve) to the direction of polarization. (**B**) Frequency-dependent amplitude and (**C**) frequency-dependent phase of the current density output. The stress here is normalized to 1 MPa. The piezoelectric coefficients d_{33} can be calculated as 117 pC N^{-1}.

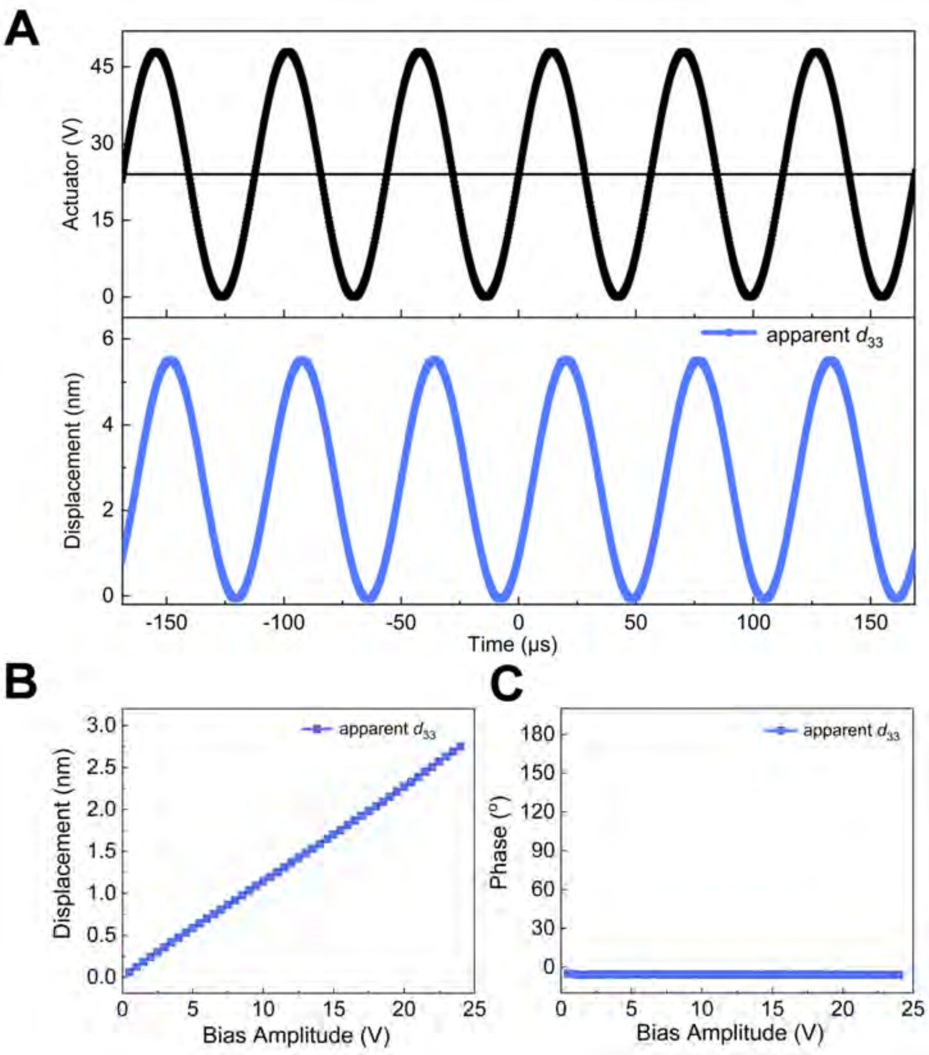

Fig. S32. Converse piezoelectric characterization of clamped $CaBi_2Nb_2O_9$ ceramic. (**A**) Waveform of applied AC voltage (top panel), and induced displacement waveform (bottom panel) for Ag/clamped $CaBi_2Nb_2O_9$/Ag capacitors, showing displacement parallel (d_{33}, blue curve) to the direction of polarization. (**B**) Voltage-dependent amplitude and (**C**) voltage-dependent phase of the displacement output, with a frequency of 17.777 kHz. The piezoelectric coefficients d_{33} can be calculated as 113 pm V^{-1}.
